# Discovery and validation of tissue-specific DNA methylation as noninvasive diagnostic markers for colorectal cancer

**DOI:** 10.1186/s13148-022-01312-9

**Published:** 2022-08-16

**Authors:** Dapeng Li, Lei Zhang, Jinming Fu, Hao Huang, Yanlong Liu, Lin Zhu, Hongru Sun, Simin Sun, Ding Zhang, Tian Tian, Fan Wang, Fulan Hu, Xiaolin Peng, Gairui Li, Liyuan Zhao, Ting Zheng, Xuan Wang, Binbin Cui, Yashuang Zhao

**Affiliations:** 1 Department of Epidemiology, School of Public Health, Harbin Medical University, Harbin, China; 2 Department of Colorectal Surgery, Harbin Medical University Cancer Hospital, Harbin Medical University, Harbin, China; 3 Department of Non-communicable Disease Prevention and Control, Shenzhen Nanshan Center for Chronic Disease, Shenzhen, China

**Keywords:** Colorectal cancer, Tissue-specific methylation, Noninvasive test, Integrative analysis

## Abstract

**Background:**

Noninvasive diagnostic markers that are capable of distinguishing patients with colorectal cancer (CRC) from healthy individuals or patients with other cancer types are lacking. We report the discovery and validation of a panel of methylation-based markers that specifically detect CRC.

**Methods:**

This was a large-scale discovery study based on publicly available datasets coupled with a validation study where multiple types of specimens from six cohorts with CRC, other cancer types, and healthy individuals were used to identify and validate the tissue-specific methylation patterns of CRC and assess their diagnostic performance.

**Results:**

In the discovery and validation cohort (*N* = 9307), ten hypermethylated CpG sites located in three genes, *C20orf194*, *LIFR*, and *ZNF304*, were identified as CRC-specific markers. Different analyses have suggested that these CpG sites are CRC-specific hypermethylated and play a role in transcriptional silencing of corresponding genes. A random forest model based on ten markers achieved high accuracy rates between 85.7 and 94.3% and AUCs between 0.941 and 0.970 in predicting CRC in three independent datasets and a low misclassification rate in ten other cancer types. In the in-house validation cohort (*N* = 354), these markers achieved consistent discriminative capabilities. In the cfDNA pilot cohort (*N* = 14), hypermethylation of these markers was observed in cfDNA samples from CRC patients. In the cfDNA validation cohort (*N* = 155), the two-gene panel yielded a sensitivity of 69.5%, specificity of 91.7%, and AUC of 0.806.

**Conclusions:**

Hypermethylation of the ten CpG sites is a CRC-specific alteration in tissue and has the potential use as a noninvasive cfDNA marker to diagnose CRC.

**Supplementary Information:**

The online version contains supplementary material available at 10.1186/s13148-022-01312-9.

## Background

Colorectal cancer (CRC) is third in terms of incidence and second in terms of mortality of cancer worldwide based on GLOBOCAN 2020 estimates [1]. Although CRC incidence has been declining, it is still the main cause of cancer deaths. The 5-year survival rate of CRC decreases significantly from 90% during the localized stage to 14% in the advanced stage. Therefore, early detection is critical to provide curable treatment and ultimately increase CRC survival rates. Multiple accepted methods are available for CRC screening, including stool-based tests and endoscopy. In addition, there is sufficient evidence that the use of these tests reduces CRC incidence and mortality, and the benefits outweigh the harms [2]. However, participant rates for these tests are low [3]. Even among the noninvasive tests of annual fecal occult blood tests, only 64.6% of patients who received a mailed reminder in the intervention group returned stool cards [4]. However, evidence suggests that 97% of participants who refused a colonoscopy were more likely to accept a noninvasive screening test, where 83% of them chose a blood test in one study [5].

DNA methylation changes are hallmarks of various cancers, highlighted by the hypermethylation of the promoter region silencing the transcription of tumor suppressor genes [6]. The use of DNA methylation-based biomarkers has been considered to be an ideal method for cancer diagnostics because of the high stability of DNA methylation over time and the easy detection using existing techniques (i.e., methylation-specific PCR) [7]. Recent studies have highlighted the potential advantages of cell-free DNA (cfDNA) methylation markers in CRC diagnosis [8]. For instance, in one study the sensitivity and specificity of cfDNA MYO1-G methylation were 84.3% and 94.5%, respectively [9]. However, only the methylation of septin-9 (*SEPT9*) was translated into clinical application for screening colon cancer approved by the FDA in 2016, with variable values of sensitivity (58–95.6%) and specificity (69–97.1%) [7]. As a tumor suppressor gene, hypermethylation of *SEPT9* was also found in the blood or tissues of other cancer type patients in addition to CRC, such as lung cancer, head and neck squamous cell cancer, and breast cancer [10–12], which could lead to a false positive diagnosis for other cancer types. As cancers originating from different tissue types may share similar methylation changes, methylation-based markers for early cancer diagnosis should have tissue-specific patterns [13]. Otherwise, it will influence the identification of the tissue origin of cancer and the selection of subsequent diagnostic methods. Hence, CRC-specific methylation markers are urgently needed.

In this study, we performed a genome-wide analysis to identify tissue-specific DNA methylation markers for the detection of CRC.

## Methods

### Study design

**Fig. 1:**
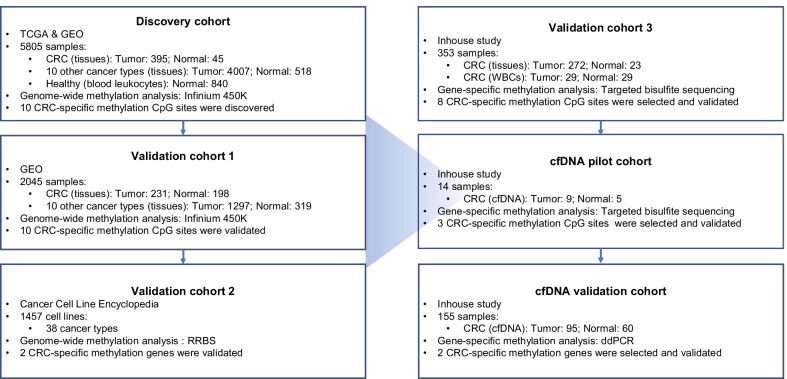
Overall workflow of this study

This study included six cohorts to discover and validate tissue-specific methylation markers of CRC (Fig. 1). Tissue-specific markers of CRC were initially discovered by comparing genome-wide methylation data from the discovery cohort (*N* = 5805) and validated in large-scale independent datasets from three validation cohorts (*N* = 3855). We further confirmed the hypermethylation of these markers in cfDNA samples of two cohorts (*N* = 160) using two PCR-based techniques. The details of the datasets are shown in Additional file 1: Table S1.

### Data source

The Cancer Genome Atlas (TCGA) for CRC and ten other types of primary solid cancer (bladder urothelial carcinoma [BLCA], breast invasive carcinoma [BRCA], esophageal carcinoma [ESCA], glioblastoma multiforme [GBM], head and neck squamous cell carcinoma [HNSC], kidney renal clear cell carcinoma [KIRC], liver hepatocellular carcinoma [LIHC], lung adenocarcinoma [LUAD], lung squamous cell carcinoma [LUSC], and uterine corpus endometrial carcinoma [UCEC]) data of Infinium HumanMethylation450 BeadChip® microarrays (Illumina Inc., San Diego, CA, USA) were downloaded using the UCSC Xena Browser (https://xena.ucsc.edu/), including 395 primary CRC tissues, 45 matched adjacent normal tissues, and 4525 tissues of ten other cancer types. The methylation level of each CpG site is represented as the beta-value (*β*), which is the ratio of the methylation intensity and the overall intensity (sum of methylation intensity and unmethylated intensity) and ranges from 0 to 1. RNA-seq gene expression data for CRC were downloaded from TCGA (https://portal.gdc.cancer.gov/) using the raw read count. Additionally, 20 methylation datasets were obtained from the Gene Expression Omnibus (GEO, https://www.ncbi.nlm.nih.gov/geo/), involving 840 samples of blood leukocytes from normal individuals (GSE40279, GSE69270), 429 samples of tissues from CRC patients (GSE42752, GSE48684, and GSE101764), and 1616 samples of tissues from ten other cancer types (GSE66695, GSE69914, GSE52826, GSE79366, GSE36278, GSE60274, GSE123678, GSE38266, GSE61441, GSE54503, GSE39279, GSE67116, and GSE93589). Furthermore, DNA methylation and gene expression parallel sequencing data from 1457 cancer cell lines for 38 cancer types were obtained from the Cancer Cell Line Encyclopedia (CCLE, http://www.broadinstitute.org/ccle/home).

### Differential analysis of DNA methylation and gene expression

Forty-five of the 395 CRC patients, who had both cancer and normal methylation profiles, were used for differential methylation analysis. CpG sites with more than 10% missing values were removed. The Bioconductor package ‘impute’ version 1.54.0 designed for imputation of microarray data was used to impute the missing data. Then, a paired t test was used to determine the differential methylation analysis, and the Benjamini‒Hochberg procedure was used to calculate the false discovery rate (FDR). Significant differentially methylated CpG sites were defined based on an FDR less than 0.05 and an absolute value of differentially methylated levels (|Δ*β*|) greater than 0.2. The CpG sites were annotated to genomic regions according to the R package ‘IlluminaHumanMethylation450kanno.ilmn12.hg19’ version 0.6.0, and annotation information of the first gene was used, while a CpG site was mapped to more than one gene. CpG sites from the X and Y chromosomes were removed. Of the 45 patients from the TCGA, 36 paired CRC and normal samples with expression data were used for differential expression analysis. The Bioconductor package ‘TCGAbiolinks’ version 2.8.3 was used to identify differentially expressed genes using raw read counts. Genes with an FDR below 0.05 and an absolute value of log_2_-fold change (|log_2_FC|) higher than 1 were identified as differentially expressed genes.

### Tissue-specific methylation marker discovery

In the discovery cohort (*N* = 5805), tissue-specific methylation markers of CRC were identified based on the following process. First, 45 paired CRC and normal samples from TCGA were used to integrate promoter methylation and gene expression to select 942 hypermethylated CpG sites (Δ*β* > 0.20, and FDR < 0.05) of downregulated genes (log_2_FC < −1, and FDR < 0.05). Second, 395 CRC and 45 normal tissues were compared to select 942 CpG sites that were still significantly differentially methylated (*P* < 0.05). Third, methylation levels were assessed in 840 samples of blood leukocytes from healthy individuals without CRC (GSE69270, GSE40279); 366 CpG sites whose average methylation levels were less than 0.1 were retained. Fourth, the 366 remaining CpG sites were filtered against 4525 samples of ten other cancer types; 356 CpG sites with average methylation levels higher than 0.1 in any group of ten other cancer types from TCGA were excluded. The remaining 10 CpG sites were potential CRC-specific methylation-based markers.

### Functional analysis

Considering the potential gene regulation function of these CpG sites, correlation analysis between the methylation of these CpG sites and their corresponding gene expression was performed using MEXPRESS (http://mexpress.be) to integrate and visualize gene expression, DNA methylation, and clinical data at the single-gene level in TCGA CRC data. Visualization of the correlation between methylation levels of each selected CpG site and neighbor sites (comethylation) was generated by the R package ‘coMET’ version 1.10.2. Furthermore, DNA methylation and gene expression data of selected markers were analyzed from 38 cancer-type cell lines from the validation cohort 2 (*N* = 1457) to verify whether these genes were specifically methylated in CRC.

### Tissue-specific methylation marker validation

TCGA data of 45 paired CRC and normal samples were used as the training set. The TCGA CRC full dataset with 395 CRC and 45 normal tissues was utilized as the internal validation set. Three independent datasets (GSE42752, GSE48684, and GSE101764) were utilized as the external validation sets. An optimized cutoff value for each CpG site at the maximal Youden’s index was calculated. At this cutoff value, a confusion matrix for each CpG site was generated in the training set and validation sets to provide performance characteristics of sensitivity and specificity. Additionally, the misclassification rate of predicting CRC in non-CRC tumors and non-CRC normal samples from ten other cancer types was also calculated at this cutoff value. To evaluate the diagnostic performance of the DNA methylation classifier, a random forest model using all selected CpG sites was built in the training set and applied to the validation set.

### Comparison with commercial methylation markers

The performance of these markers in the tissue was compared with three methylation-based biomarkers from three commercially available assays (the N-myc downregulated gene 4 [*NDRG4*], the bone morphogenetic protein 3 [*BMP3*] biomarkers of *Cologuard* and the *SEPT9* biomarker of *Epi proColon* and *Epi proColon/ColoVantage*). The methylation data of the CpG sites that were covered or surrounded by primer sequences of the three assays and other published articles were extracted [14–26].

### Biospecimen sources

To validate the findings from publicly available datasets, we carried out diagnostic test evaluation in three cohorts: the validation cohort 3 from 272 CRC tissues, 23 adjacent normal tissues, 29 CRC white blood cell samples, and 29 healthy control white blood cell samples; a cfDNA pilot cohort from 9 CRC cfDNA samples and 5 healthy control cfDNA samples; and a cfDNA validation cohort from 95 CRC cfDNA samples and 60 healthy control cfDNA samples. All CRC samples were collected at Harbin Medical University Cancer Hospital, from diagnosed patients who underwent a colonoscopy before neoadjuvant chemotherapy. All healthy control samples were obtained from volunteers without CRC or colorectal adenoma who underwent colonoscopy. Informed consent was obtained from all study participants. The approval for the research on human subjects was obtained from the Medical Ethics Committee of Harbin Medical University.

### DNA extraction and bisulfite conversion

The genomic DNA from tissues and white blood cells were extracted with the classic phenol‒chloroform procedure and the QIAamp DNA Blood Mini Kit (QIAGEN GmbH, Hilden, Germany) according to the manufacturer’s instructions. Samples of cfDNA were isolated from 4 mL of plasma using the QIAamp Circulating Nucleic Acid Kit (QIAGEN GmbH, Hilden, Germany) according to the recommended protocol. The DNA samples were bisulfite-converted with the EZ DNA Methylation-Gold™ Kit (Zymo Research, Irvine, CA, USA) according to the manufacturer’s protocol.

### DNA methylation detection

We performed methylation analysis using two PCR-based techniques for this study. We first developed and performed targeted bisulfite sequencing analysis in the validation cohort 3 (*N* = 354) and the cfDNA pilot cohort (*N* = 14). DNA methylation level was quantified using MethylTarget sequencing (Genesky Biotechnologies Inc., China). Briefly, methylation levels of the target regions were tested using a two-step PCR approach. Sequencing was performed on the Illumina HiSeq 2000 platform in 150 bp paired-end mode (Additional file 1: Supplementary methods). In the cfDNA validation cohort (*N* = 155), the methylation levels of the selected markers were detected in cfDNA samples from CRC patients and healthy controls using droplet Digital PCR (ddPCR). The converted DNA was subjected to ddPCR using the designed primers and the optimized conditions on the QX200 ddPCR System (Additional file 1: Supplementary methods).

### Statistical analysis

All statistical tests were two-sided, and a *P* value < 0.05 was considered statistically significant unless otherwise specified. All analyses were conducted in the R version 3.5.1. All the genomic coordinates corresponded to the human reference genome version GRCh37/hg19. The methylation levels of the two groups were compared by the Student’s t test or the paired *t* test. The correlation between the methylation level of the CpG sites and the corresponding gene expression level was assessed by the Pearson correlation coefficient. The comethylation at the adjacent CpG sites was analyzed using the Spearman correlation. ROC curves were used to determine the optimal cutoff value for each candidate CpG site at the maximal Youden’s index. The random forest model was built using the R package ‘caret’ version 6.0-80. Sensitivity, specificity, accuracy, and AUC were used to evaluate the diagnostic performance of the individual CpG sites and models. A sample size of 46 participants for each group of CRC and healthy control was estimated to be able to provide a power of 80% in a pre-estimate AUC of 0.80 and AUC with 95% confidence the degree of estimate about 0.10 [27].

## Results

### Integrative analysis of DNA methylation and gene expression

**Fig. 2:**
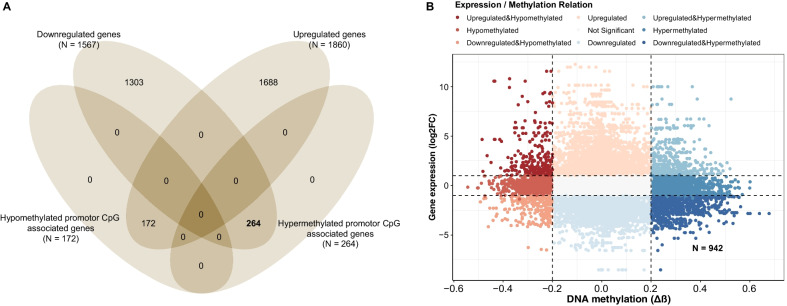
Relationship between DNA methylation of promoter CpG sites and gene expression. **A** A four-way Venn diagram shows intersection of genes containing differentially methylated promoter CpG sites and differentially regulated genes. **B** Starburst plot shows integrating analysis of gene expression changes and DNA methylation changes of promoter CpG sites. Dots represent individual promoter CpG and the corresponding gene expression. Red dots indicate a total of 942 hypermethylated promoter CpG probes located in 264 downregulated genes

With a threshold of |Δ*β*|> 0.20 and an FDR < 0.05, a total of 42,102 differentially methylated CpG sites were identified in the 45 paired TCGA CRC datasets, which were mapped to 7087 genes (Additional file 1: Fig. S1). The genomic location of the differentially methylated CpG sites was analyzed. Globally, we identified 19,183 hypermethylated CpG sites located in 2977 known genes and 22,919 hypomethylated CpG sites associated with 5487 known genes. By the intersection of the differential methylation and gene expression, a total of 1468 genes were differentially methylated and differentially expressed, where 264 downregulated genes had 942 hypermethylated CpG sites in the promoter region (Fig. 2).

### Discovery of tissue-specific methylation markers of CRC

**Fig. 3:**
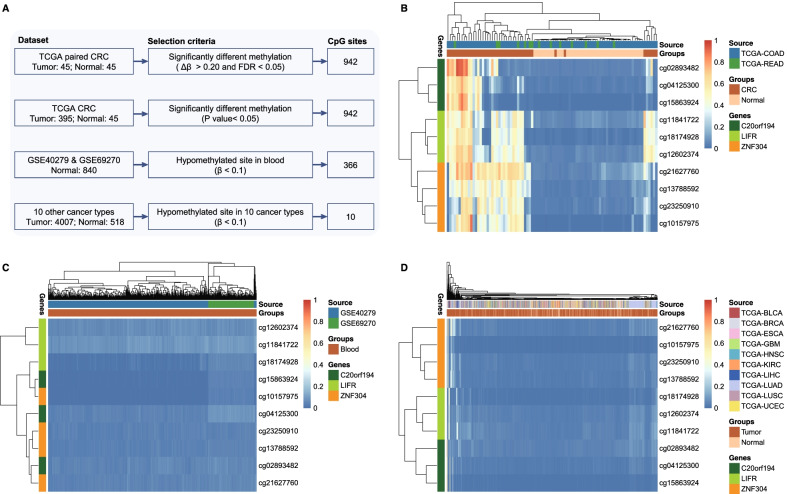
Discovery of specific methylation-based markers. **A** A process for identification of candidate DNA methylation-based markers of CRC. **B** Unsupervised hierarchical clustering of ten methylation-based markers selected for the diagnosis of CRC in the 45 TCGA paired CRC samples. **C** Unsupervised hierarchical clustering of ten methylation-based markers in 840 blood leukocytes of healthy individuals. **D** Unsupervised hierarchical clustering of ten methylation-based markers in 4525 tumor and normal tissues of ten other cancer types

A total of 942 CpG sites were identified by integrative analysis of promoter methylation and gene expression, which were still significantly differentially methylated between the 395 CRC tissues and the 45 normal tissues (Fig. 3B). Then, 366 CpG sites were selected after removing 576 hypermethylated CpG sites (mean *β* > 0.1) in 840 normal blood samples (Fig. 3C). Finally, ten CRC-specific CpG sites were obtained by removing hypermethylated CpG sites (mean *β* > 0.1) in 4525 samples of the ten other cancer types (Fig. 3D). The ten CRC-specific CpG sites were annotated to three genes (Additional file 1: Table S2): chromosome 20 open reading frame 194 (*C20orf194*; cg04125300, cg15863924, and cg02893482), LIF receptor alpha (*LIFR*; cg18174928, cg12602374, and cg11841722), and zinc finger protein 304 (*ZNF304*; cg23250910, cg10157975, cg13788592, and cg21627760). These CpG sites in the same gene were located in neighboring regions, and 9 out of 10 were located in CpG islands (CGIs).

### Functional analysis of tissue-specific methylation markers

With the underlying assumption that hypermethylated CpG sites in promoter CGIs can play a role in gene silencing, the correlation between DNA methylation and gene expression was investigated (Additional file 1: Figs. S2, S3, S4). All methylation of the ten CpG sites was significantly negatively correlated with gene expression, with Pearson correlation coefficients (*r*) of − 0.214 to − 0.642. Furthermore, the Spearman correlation method was used to study the pattern of comethylation. The results showed a highly consistent methylation status of the selected CpG sites with neighboring CpG sites in the same promoter region. The results from the CCLE support that the selected genes *C20orf194* and *ZNF304* (*LIFR* without data) were specifically hypermethylated and downregulated in CRC cell lines compared to those of other cancer types (Additional file 1: Fig. S5).

### Evaluation and validation of the performance of tissue-specific methylation markers

**Fig. 4:**
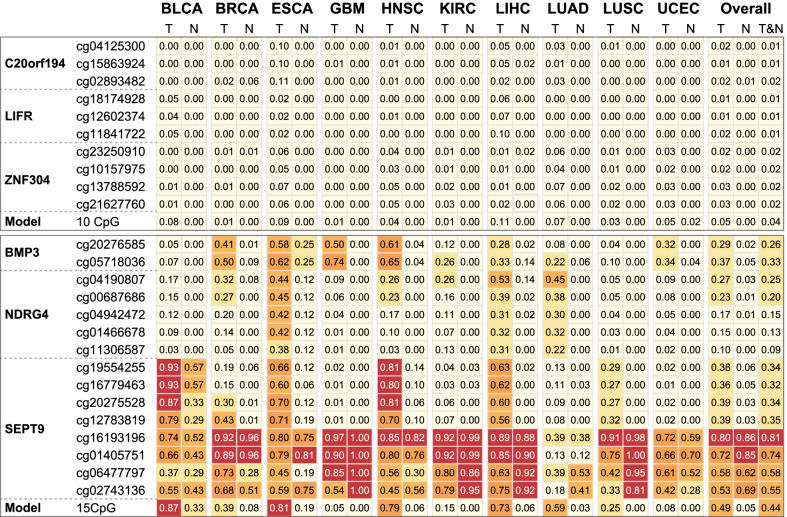
Comparison of tissue-specific methylation markers with previously commercial methylation markers. Heatmap shows misclassification rate of 10 CpG sites from our study and 15 CpG sites from three commercial biomarkers in distinguishing CRC samples from ten other cancer types in TCGA dataset. Numbers represent the misclassification rate of predicted CRC in non-CRC tumor (T) and non-CRC normal (N) samples

The differences in methylation levels of the ten CpG sites between CRC samples and normal samples were similar in four different CRC datasets (Additional file 1: Table S3 and Fig. S6). By using the optimal cutoff value for individual CpG sites, the observed sensitivity of an individual CpG for CRC detection ranged from 33.9 to 86.4% and the specificity ranged from 87.8 to 100% (Additional file 1: Table S4). To evaluate the combined performance of these hypermethylated CpG sites, 45 paired CRC tissues and normal tissues from TCGA were used as a training dataset to build a random forest model. In the internal validation datasets, the accuracy rate was 93.6% (95% CI 90.9–95.7%; Table 1) with an AUC value of 0.996 (Additional file 1: Fig. S6). The random forest model using the ten CpG sites achieved high predictive performances in three external validation datasets (Table 1). The accuracy rates were more than 85%, with values of 85.7% (95% CI 74.6–93.3%) in GSE42752, 85.7% (95% CI 77.5–91.8%) in GSE48684, and 94.3% (95% CI 90.7–96.8%) in GSE101764. The corresponding AUC values were above 0.94 in all three datasets (Additional file 1: Fig. S6). With this model, only 4.1% (187 of 4525) of samples from the ten other cancer types in TCGA were misclassified into the CRC group (Fig. 4), which was consistent with the GEO datasets (misclassification rate = 6.9%, 111 of 1616; Additional file 1: Table S5).

**Table 1:** Confusion matrix of prediction performance of random forest model using 10 CRC-specific methylation CpG sites in distinguishing CRC from normal samples

Validation dataset	TCGA	GSE42752	GSE48684	GSE101764
Accuracy (95% CI)	0.936 (0.909, 0.957)	0.857 (0.746, 0.933)	0.857 (0.775, 0.918)	0.943 (0.907, 0.968)
Balanced accuracy	0.965	0.869	0.839	0.938
Sensitivity	0.929	0.909	0.922	0.902
Specificity	1.000	0.829	0.756	0.973
Kappa	0.728	0.701	0.693	0.882

### Comparison with previously commercial methylation markers

Then, a comparison with three methylation-based biomarkers (*NDRG4*, *BMP3* and *SEPT9*) of commercially available assays was performed (Additional file 1: Table S2). Compared with our selected CpG sites, individual CpG sites from three commercial biomarkers had similar performance in discriminating CRC from normal colorectal samples (Additional file 1: Table S3, Table S4). However, poor performance was observed in predicting tumor and normal samples from the ten other cancer types (Fig. 4; Additional file 1: Table S5) because these CpG sites had similar patterns between CRC and the ten other cancer types (Additional file 1: Fig. S6). The random forest model using the 15 CpG sites of commercial methylation markers had a higher performance (accuracy rate: 98.4%) in the internal validation of the TCGA CRC dataset, while it achieved a lower performance (accuracy rate: 75.2–92.0%) in the three external validation datasets (Additional file 1: Table S6). Importantly, a large percentage of tumor and normal samples from the ten other cancer types in both TCGA (43.8%, 1980 of 4525; Fig. 4) and GEO (41.0%, 663 of 1616; Additional file 1: Table S5) were misclassified into the CRC group using the commercial methylation markers.

### Validation in samples of tissue and white blood cell in an in-house study

**Fig. 5:**
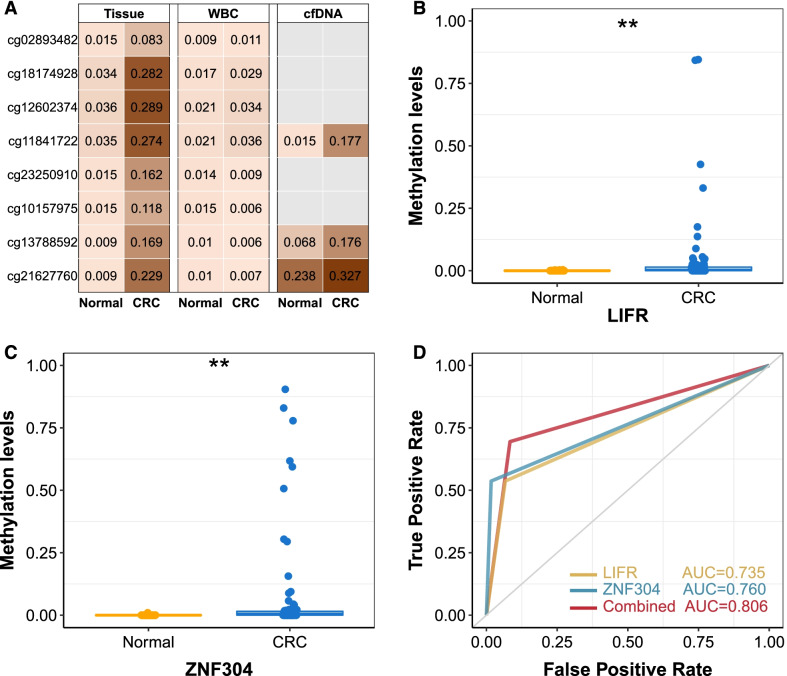
Validation of specific methylation-based markers in in-house study. **A** Validation of the methylation markers using target bisulfite sequencing array in validation cohort 3 and cfDNA pilot cohort. Numbers represent average methylation levels of CpG sites in multiple specimens. **B** and **C** Validation of the methylation markers using Droplet Digital PCR in cfDNA validation cohort. **P < 0.05. **D** ROC curves for tissue-specific methylation markers in the cfDNA validation cohort

To verify findings from public datasets, we performed targeted bisulfite sequencing analysis in tissues and white blood cells in the validation cohort 3 (Additional file 1: Fig. S7 and Table S7). Differential methylation analysis of these markers (8 CpG sites in the *LIFR* and *ZNF304* genes were selected) showed that all 8 CpG sites were significantly hypermethylated in 272 CRC tissues compared to 23 normal tissues (Fig. 5A). ROC analysis revealed that 8 CpG sites yielded AUC values of 0.645–0.896 to distinguish CRC tissues and normal tissues (Additional file 1: Fig. S8). By using the optimal cutoff value, the observed sensitivity and specificity of an individual CpG site ranged between 47.4 and 81.6% and 95.7 and 100.0%, respectively. Moreover, methylation levels of 8 CpG sites were very low in white blood cell samples from CRC patients (*N* = 29) and healthy controls (*N* = 29), consistent with the findings from public datasets.

### Validation in cfDNA samples

To test whether this tissue-specific methylation could be a noninvasive marker, we developed and applied targeted bisulfite sequencing analysis (3 CpG sites in the *LIFR* and *ZNF304* genes were selected) in the cfDNA pilot cohort with 9 cfDNA samples from CRC patients and 5 cfDNA samples from healthy controls. The mean methylation value of 3 CpG sites was higher in the CRC patients than in the healthy controls (Fig. 5A; Additional file 1: Fig. S7). To further assess the clinical applications of these markers, we detected the methylation status of *LIFR* (Fig. 5B) and *ZNF304* (Fig. 5C) in the cfDNA validation cohort with 95 cfDNA samples from CRC patients and 60 cfDNA samples from healthy controls using ddPCR. We detected *LIFR* hypermethylation in 51 cfDNA samples from CRC patients and 4 healthy controls. *ZNF304* hypermethylation was observed in 51 CRC patients and 1 healthy control. The two-gene panel yielded a sensitivity of 69.5% (66 of 95), a specificity of 91.7% (55 of 60), and an AUC of 0.806 (Fig. 5D). The two-gene panel was more sensitive in advanced-stage CRC, with a sensitivity of 54.5% in early-stage CRC and 72.4% in advanced-stage CRC.

## Discussion

cfDNA carries cancer-specific genetic and epigenetic aberrations and thus can be used as a noninvasive diagnostic marker for cancer [28]. Our proof-of-concept study demonstrated that it was feasible to discover CRC-specific markers by genome-wide methylation analysis of multiple cancers coupled with validation using cfDNA samples. The current findings were limited by the lack of validation in cfDNA samples from other cancer types and colorectal adenoma.

A growing number of studies have examined cfDNA methylation as a potential diagnostic biomarker for cancers, including CRC, with the following strategies. The markers were discovered by differential methylation analysis between CRC tissues and normal tissues, and the diagnostic ability of cfDNA was further assessed. Although these strategies have identified numerous methylation-based markers with high sensitivity, the specificity of the tumor-derived signal is doubtful. These strategies run counter to the well-established principle of ctDNA methylation as a diagnostic marker in that cancers, including CRC, should have a unique pattern of DNA methylation. Previously, cfDNA methylation markers for CRC detection were limited by high noise signals from other cancer types. For example, our systematic analysis suggested that using *SEPT9* for the detection of CRC was limited by high misclassification rates in other cancer types. Correspondingly, a study found plasma *SEPT9* methylation in HNSCC patients [29]. In addition, 94 of 98 patients with liver cancer tested positive by the *SEPT9* array [30]. The performance of this array in distinguishing other diseases was limited because the positive rate was 17.28% (33 of 191) in subjects with nontumor chronic conditions and 41.62% (72 of 173) in subjects with non-CRC-related cancers [31]. Recently published studies have shown that unique DNA methylation alterations can be found in a particular tissue and have used genome-wide methylation approaches in cfDNA to infer the contributions of different tissues [32–36]. Therefore, the use of tissue-specific methylation in cfDNA could be used to trace the tissue of origin from plasma DNA, which has the potential to be a diagnostic biomarker for various diseases, including cancers. However, a more cost-effective method is required for the use of tissue-specific methylation patterns for the diagnosis of CRC; that is, a small number of diagnostic markers need to be identified for clinical translation. Using large public methylome databases, we present a strategy that searches for unique methylation markers derived from CRC by controlling for the methylation patterns of other cancer types. We also controlled the methylation levels of the markers in white blood cells, as the largest proportion of cfDNA originates from white blood cells. Our development strategy ensured that the markers were highly specific for the detection of CRC. Importantly, we performed blood tests to assess tissue-specific methylation patterns in cfDNA using two cost-effective PCR-based technologies and found that a few markers could diagnose CRC with high specificity.

To the best of our knowledge, the ten CpG sites discovered in our study have not been previously reported for the diagnosis of CRC. Only a few studies have reported that two corresponding genes, *LIFR* and *ZNF304*, are hypermethylated in CRC tissues or cell lines using different experimental methods. Cho YG et al. observed a higher frequency (65%, 52 of 80) of promoter hypermethylation of *LIFR* in colon cancer samples than in matched normal tissues (5%, 4 of 80) and colon normal mucosa tissues (0%, 0 of 13) of noncancer patients using quantitative methylation-specific PCR (qMSP) [37]. This study also found downregulation of both *LIFR* mRNA and protein expression in CRC tissues. Jeon K et al. tested 46 cancer cell lines using targeted bisulfite PCR sequencing involving the five cancer types of colon, biliary tract, liver, lung, and stomach and found that a CpG site (chr5: 38557143) in *LIFR*, which was located within two selected CpG sites of cg18174928 (chr5: 38557085) and cg12602374 (chr5: 38557162) in our studies, was heavily methylated in colon cancer cells only but not in other cancer cell lines [38]. *ZNF304* acts as a transcriptional regulator and plays a role in gene silencing. We found that *ZNF304* was hypermethylated and downregulated in CRC tissues. This result is supported by a study reporting that *ZNF304* was hypermethylated in CRC tissues using Infinium 27 K and was validated by MSP in CRC tissues, adjacent normal tissues, normal colon cells, and CRC cell lines [39]. The mRNA expression of *ZNF304* was restored after demethylating treatment with 5-aza-2'-deoxycytidine (5-aza-dC) and vincristine in CRC cells [39]. These studies enhanced the power of our analysis in identifying novel CRC-specific methylation markers.

Our study provides proof of concept for the utility of large-scale genome-wide methylation data from multiple cancer types for studying tissue-specific methylation for the diagnosis of CRC, and these findings were extended to noninvasive approaches. However, our study also has some limitations. First, the validation study was a single-center design with moderately sized cfDNA samples. Second, the sensitivities of these markers were not as high as expected, and in the future, we will optimize experimental conditions and build an analytic operating procedure to improve and evaluate their diagnostic performance in large and multiple cohorts. Third, although our study provides convincing evidence for the clinical feasibility of our methylation markers to specifically diagnose CRC, the specificity should be compared with that of commercial markers in the cfDNA validation cohort with different cancer types. Finally, our markers were hypermethylated in the tissue samples from colorectal adenoma patients (Additional file 1: Fig. S9); however, whether these markers can serve as noninvasive biomarkers for colorectal adenomas needs to be evaluated.

## Conclusions

In conclusion, using genome-wide methylation data and samples of tissues and cfDNA, we identified and validated ten tissue-specific methylation sites as noninvasive markers that could detect CRC with high levels of specificity.

## Supplementary Information

**Additional file 1.** **Fig. S1**: Integrative analysis of DNA methylation and gene expression. **Fig. S2**: Correlation analysis of selected CpG sites in relation to gene expression and methylation of neighbor CpG sites in C20orf194. **Fig. S3**: Correlation analysis of selected CpG sites in relation to gene expression and methylation of neighbor CpG sites in LIFR. **Fig. S4**: Correlation analysis of selected CpG sites in relation to gene expression and methylation of neighbor CpG sites in ZNF304. **Fig. S5**: Methylation and expression levels of C20orf194 and ZNF304 in Cancer Cell Line Encyclopedia. **Fig. S6**: Validation of 10 CRC-specific methylation CpG sites of our study and 15 CpG sites of three commercial biomarkers. **Fig. S7**: Methylation profiles of (A) C20orf194, (B) LIFR and (C) ZNF304 in an in-house study using targeted bisulfite sequencing analysis. **Fig. S8**: Receiver operating characteristic curve analyses of 8 CpG sites for discriminating colorectal cancer tissues and adjacent normal tissues in the in-house study. **Fig. S9**: Validation of 10 CRC-specific methylation CpG sites of our study in tissues of colorectal adenomas and normal tissues. **Table S1**: Datasets used for discovery and validation of CRC-specific methylation markers. **Table S2**: Genomic information of 10 CRC-specific methylation CpG sites of our study and 15 CpG sites from three commercial biomarkers. **Table S3**: Differential methylation analysis of 10 CRC-specific methylation CpG sites of our study and 15 CpG sites of three commercial biomarkers in CRC samples and normal samples. **Table S4**: Prediction performance of 10 CRC-specific methylation CpG sites of our study and 15 CpG sites of three commercial biomarkers. **Table S5**: Misclassification rate of 10 CRC-specific methylation CpG sites of our study and 15 CpG sites of three commercial biomarkers in GEO dataset. **Table S6**: Confusion matrix of random forest model using 15 CpG sites from three commercial biomarkers in distinguishing CRC samples from normal samples. **Table S7**: Sequences of primers and products of targeted bisulfite sequencing array and Droplet Digital PCR.

## Data Availability

The datasets supporting the conclusions of this article are available in the Gene Expression Omnibus repository (https://www.ncbi.nlm.nih.gov/geo/) and UCSC Xena Browser (TCGA, https://xena.ucsc.edu/). The data and code used in the present study are available in https://doi.org/10.5281/zenodo.6815297.

## Abbreviations

CRC: Colorectal cancer
cfDNA: Cell-free DNA
BLCA: Bladder urothelial carcinoma
BRCA: Breast invasive carcinoma
ESCA: Esophageal carcinoma
GBM: Glioblastoma multiforme
HNSC: Head and neck squamous cell carcinoma
KIRC: Kidney renal clear cell carcinoma
LIHC: Liver hepatocellular carcinoma
LUAD: Lung adenocarcinoma
LUSC: Lung squamous cell carcinoma
UCEC: Uterine corpus endometrial carcinoma
TCGA: The Cancer Genome Atlas
GEO: Gene Expression Omnibus
FDR: False discovery rate
CCLE: Cancer Cell Line Encyclopedia
ROC: Receiver operating characteristic curve
AUC: Area under ROC
ddPCR: Droplet Digital PCR
CGI: CpG islands
qMSP: Quantitative methylation-specific PCR
