# Supplementary material for: Discovery and validation of tissue-specific DNA methylation as noninvasive diagnostic markers for colorectal cancer
Source: Clin Epigenetics. 2022 Aug 16;14:102. doi: 10.1186/s13148-022-01312-9 (PMC9382793; doi:10.1186/s13148-022-01312-9)
Supplement: Supplementary file 1 — Additional file 1. Fig. S1: Integrative analysis of DNA methylation and gene expression. Fig. S2: Correlation analysis of selected CpG sites in relation to gene expression and methylation of neighbor CpG sites in C20orf194. Fig. S3: Correlation analysis of selected CpG sites in relation to gene expression and methylation of neighbor CpG sites in LIFR. Fig. S4: Correlation analysis of selected CpG sites in relation to gene expression and methylation of neighbor CpG sites in ZNF304. Fig. S5: Methylation and expression levels of C20orf194 and ZNF304 in Cancer Cell Line Encyclopedia. Fig. S6: Validation of 10 CRC-specific methylation CpG sites of our study and 15 CpG sites of three commercial biomarkers. Fig. S7: Methylation profiles of (A) C20orf194, (B) LIFR and (C) ZNF304 in an in-house study using targeted bisulfite sequencing analysis. Fig. S8: Receiver operating characteristic curve analyses of 8 CpG sites for discriminating colorectal cancer tissues and adjacent normal tissues in the in-house study. Fig. S9: Validation of 10 CRC-specific methylation CpG sites of our study in tissues of colorectal adenomas and normal tissues. Table S1: Datasets used for discovery and validation of CRC-specific methylation markers. Table S2: Genomic information of 10 CRC-specific methylation CpG sites of our study and 15 CpG sites from three commercial biomarkers. Table S3: Differential methylation analysis of 10 CRC-specific methylation CpG sites of our study and 15 CpG sites of three commercial biomarkers in CRC samples and normal samples. Table S4: Prediction performance of 10 CRC-specific methylation CpG sites of our study and 15 CpG sites of three commercial biomarkers. Table S5: Misclassification rate of 10 CRC-specific methylation CpG sites of our study and 15 CpG sites of three commercial biomarkers in GEO dataset. Table S6: Confusion matrix of random forest model using 15 CpG sites from three commercial biomarkers in distinguishing CRC samples from normal samples. Table S7 [file 13148_2022_1312_MOESM1_ESM.docx]

**SUPPLEMENTARY MATERIAL**

# Supplementary methods

1. **Targeted bisulfite sequencing**

The next generation sequencing-based targeted bisulfite sequencing includes the following steps: Primers (**Table S7**) were designed using primer3 (http://primer3.ut.ee/) based on the genomic position of CpG sites from public available datasets. A two-step PCR approach was performed for each bisulfite-converted DNA sample, with a multiplexed PCR amplification followed by an index PCR. Paired-end sequencing (2 × 150 bp) was performed on the Illumina HiSeq platform (Illumina, San Diego, CA, USA). After quality control and filtering, paired reads were merged using FLASH, then were mapped to the reference genome of the human GRCh37/hg19 using blast + . Methylation level of each CpG sites were calculated as methylated reads counts divided by total read counts.

**2. Droplet Digital PCR**

The QX200™ Droplet Digital™ PCR System (Bio-Rad) was used to measure the methylation levels in cfDNA. Primers and probes were designed using ABI Primer Express software (version 3.0.1) and synthesized by Sangon Biotech (Shanghai). The ddPCR reaction mixture consisted of 10 μl ddPCR Supermix for Probes (No dUTP, Cat#1863024), and approximately 5-6 μl bisulfite-converted DNA template, primers (0.9 μM) and probes (0.25 μM), and RNase-Free water, in a final volume of 21 μl. The following cycling conditions were used: 95 ℃ for 10 min, followed by 40 cycles at 94 ℃ for 10 s and 60 ℃ for 60 s (at ramp rate 2 ℃/s), and finally 98 ℃ for 10 min. After thermo cycling, the 96-well plate was loaded into a QX200 droplet reader for droplet amplitude reading. Data from the QX200 Droplet Reader was analyzed in QuantaSoft version 1.7.4 (Bio-Rad). With a methylated DNA control, a unmethylated DNA control, a RNase-Free water control, and a non-template control (NTC) in the 96-well plate, the threshold was determined for distinguishing between positive and negative droplets. Samples with a total number of droplets less than 8000 are excluded. Methylation level = methylated copies / (methylated copies + non-methylated copies).

# Supplemental Figures

**
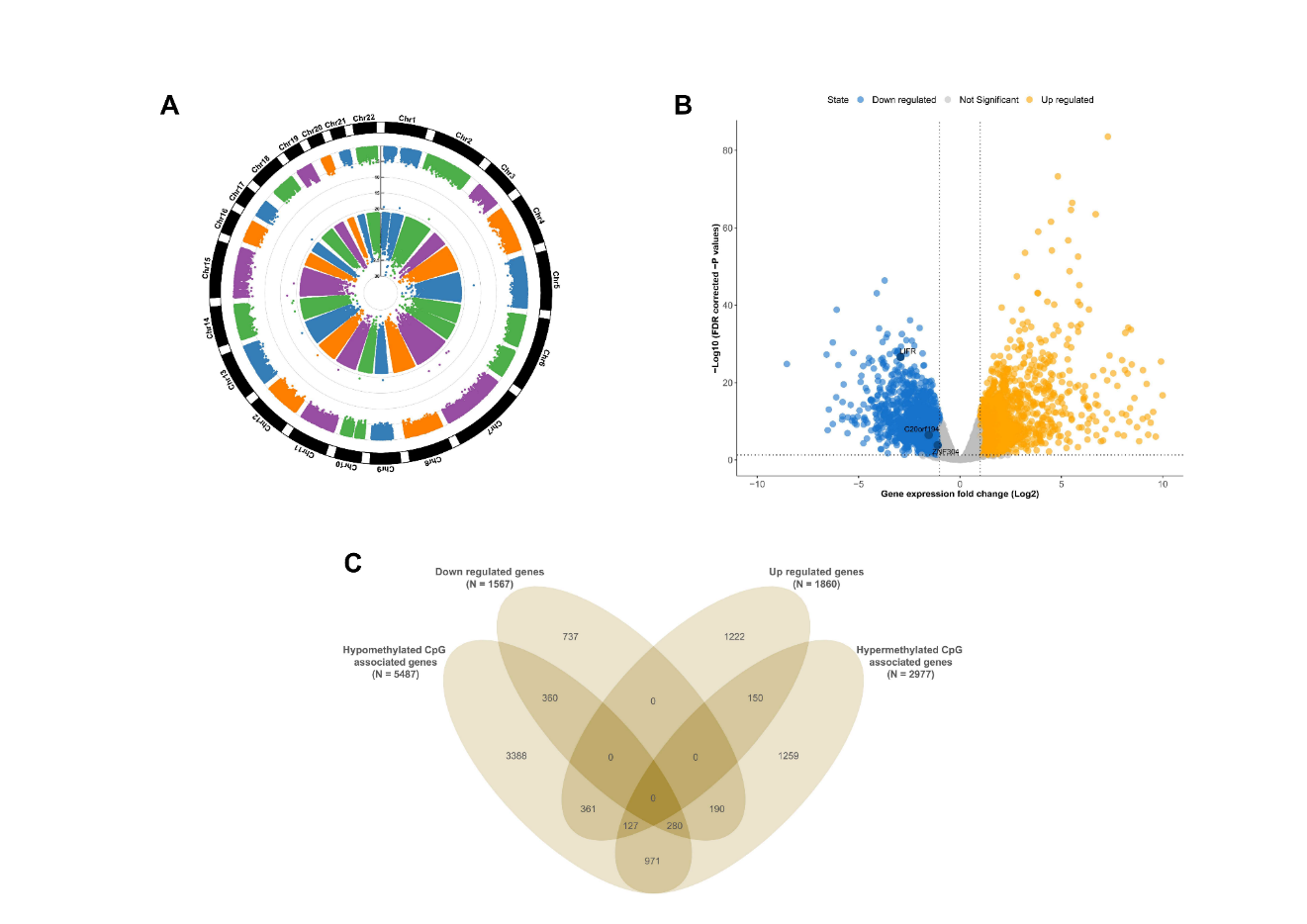
**

## Fig. S1. Integrative analysis of DNA methylation and gene expression.

**(A)** Differential analysis of DNA methylation in TCGA 45 paired CRC samples. Manhattan plot shows the chromosome (chr) and positional (pos) information of each CpG sites. Dots in inner ring represents log_10_ FDR, dots in outer ring represents log_10_ |Δβ|. **(B)** Differential analysis of gene expression in TCGA 36 paired CRC samples. A volcano plot showing the -log_10_FDR against the log_2_ fold change for each gene. **(C)** Integrative analysis of DNA methylation and gene expression. A four-way Venn diagram shows intersection of genes containing differential methylated CpG sites and differential regulated genes.

**
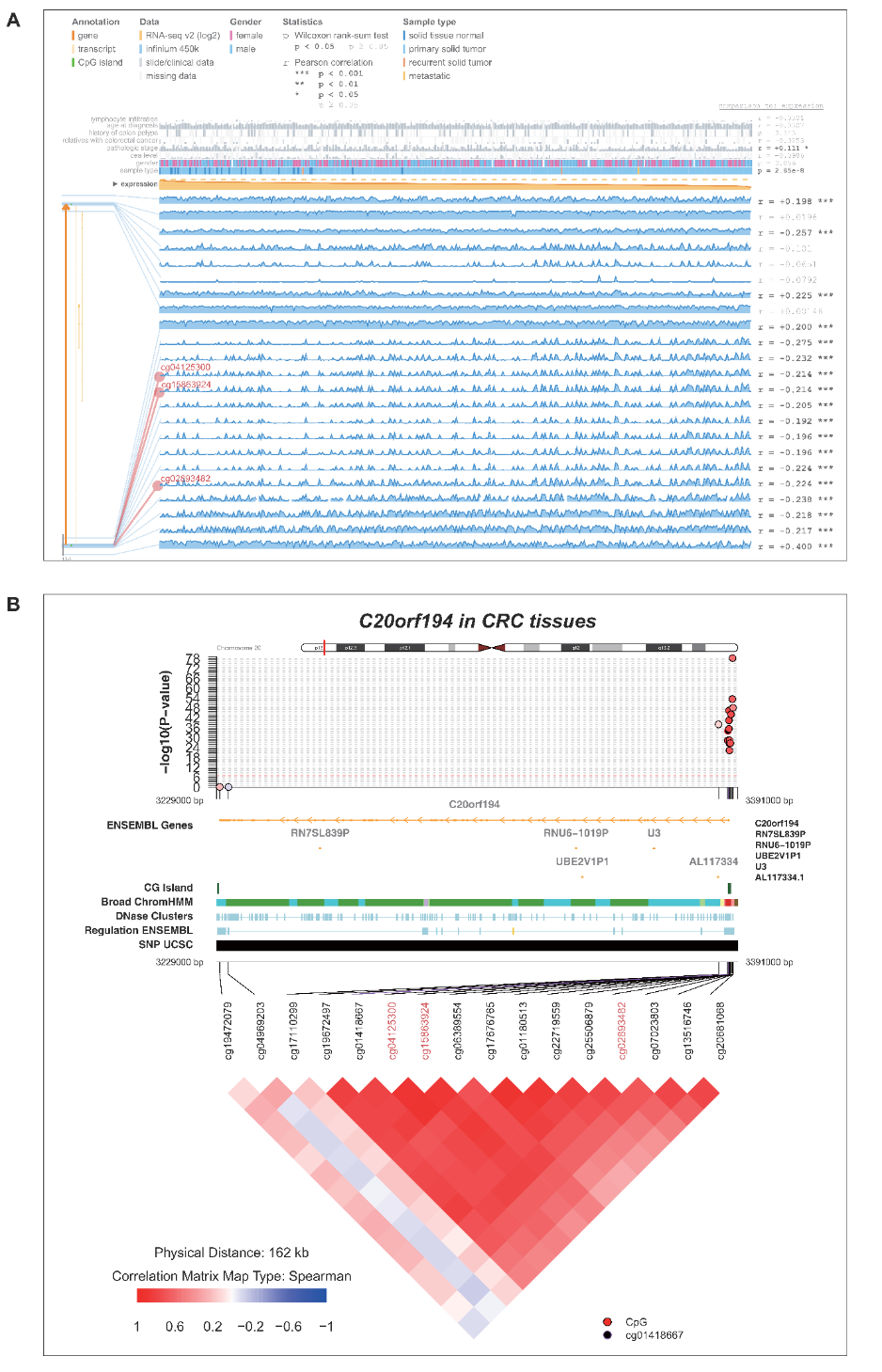
**

## **Fig. S2. Correlation analysis of selected CpG sites in relation to gene expression and methylation of neighbor CpG sites in C20orf194.**

**(A)** Correlation analysis between methylation levels of selected CpG sites at C20orf194 and relative gene expression. Visualization of the TCGA data for C20orf194 in CRC was using MEXPRESS. The samples are ordered by their expression value. The direction of correlation between C20orf194 expression and methylation is confirmed by the Pearson correlation coefficients. **(B)** Regional plot of co-methylation patterns at the C20orf194 gene in TCGA CRC tissues. The upper plot shows the results (-log_10_FDR) of differential methylation between 395 CRC samples and 45 normal samples. The middle panel shows annotation tracks. The lower panel shows Spearman correlation analysis between CpG sites in the genomic region of C20orf194.

**
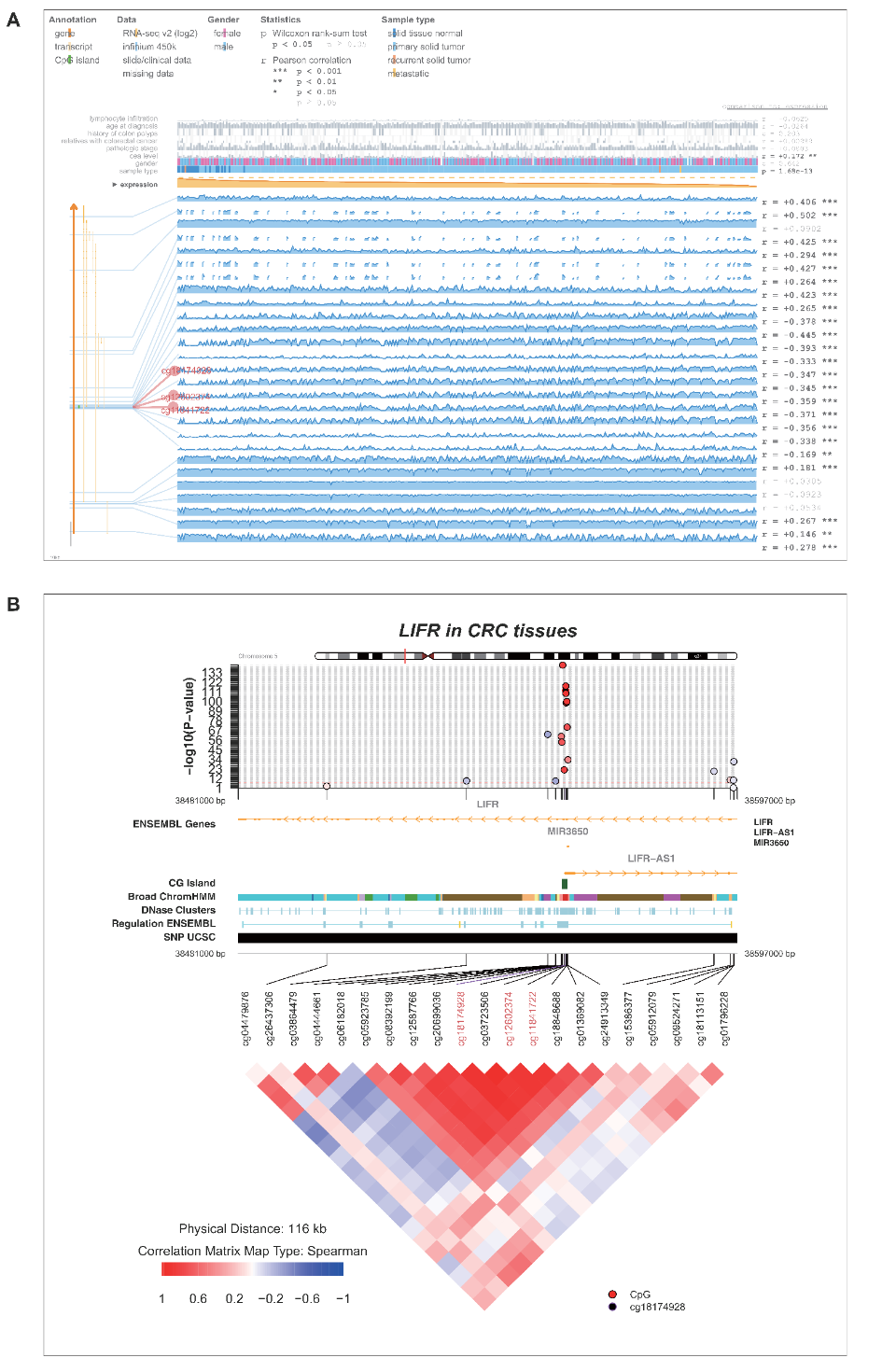
**

## **Fig. S3. Correlation analysis of selected CpG sites in relation to gene expression and methylation of neighbor CpG sites in LIFR.**

**(A)** Correlation analysis between methylation levels of selected CpG sites at LIFR and relative gene expression. Visualization of the TCGA data for LIFR in CRC was using MEXPRESS. The samples are ordered by their expression value. The direction of correlation between LIFR expression and methylation is confirmed by the Pearson correlation coefficients. **(B)** Regional plot of co-methylation patterns at the LIFR gene in TCGA CRC tissues. The upper plot shows the results (-log10FDR) of differential methylation between 395 CRC samples and 45 normal samples. The middle panel shows annotation tracks. The lower panel shows Spearman correlation analysis between CpG sites in the genomic region of LIFR.


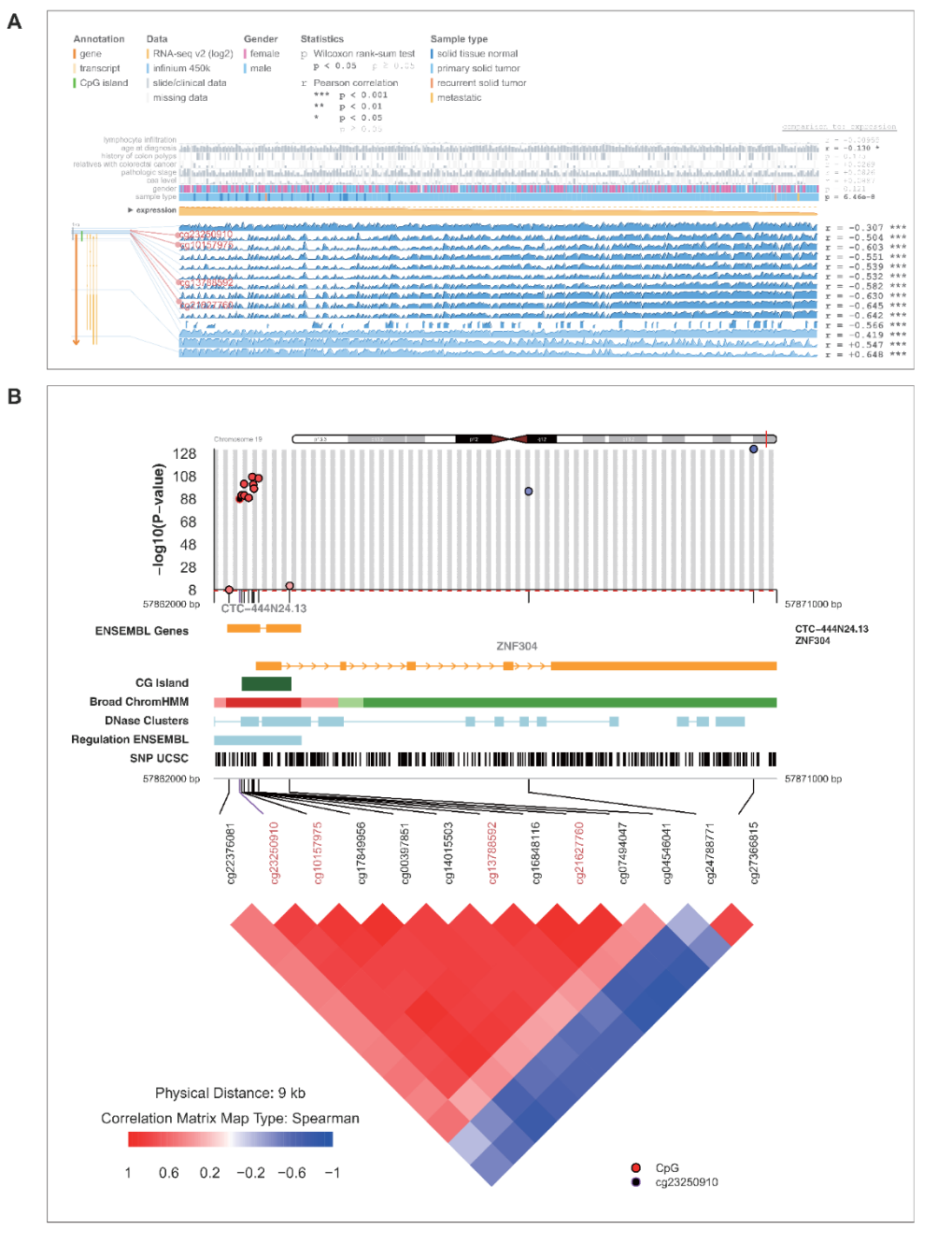


## **Fig. S4. Correlation analysis of selected CpG sites in relation to gene expression and methylation of neighbor CpG sites in ZNF304.**

(A) Correlation analysis between methylation levels of selected CpG sites at ZNF304 and relative gene expression. Visualization of the TCGA data for ZNF304 in CRC was using MEXPRESS. The samples are ordered by their expression value. The direction of correlation between ZNF304 expression and methylation is confirmed by the Pearson correlation coefficients. (B) Regional plot of co-methylation patterns at the ZNF304 gene in TCGA CRC tissues. The upper plot shows the results (-log_10_FDR) of differential methylation between 395 CRC samples and 45 normal samples. The middle panel shows annotation tracks. The lower panel shows Spearman correlation analysis between CpG sites in the genomic region of ZNF304.


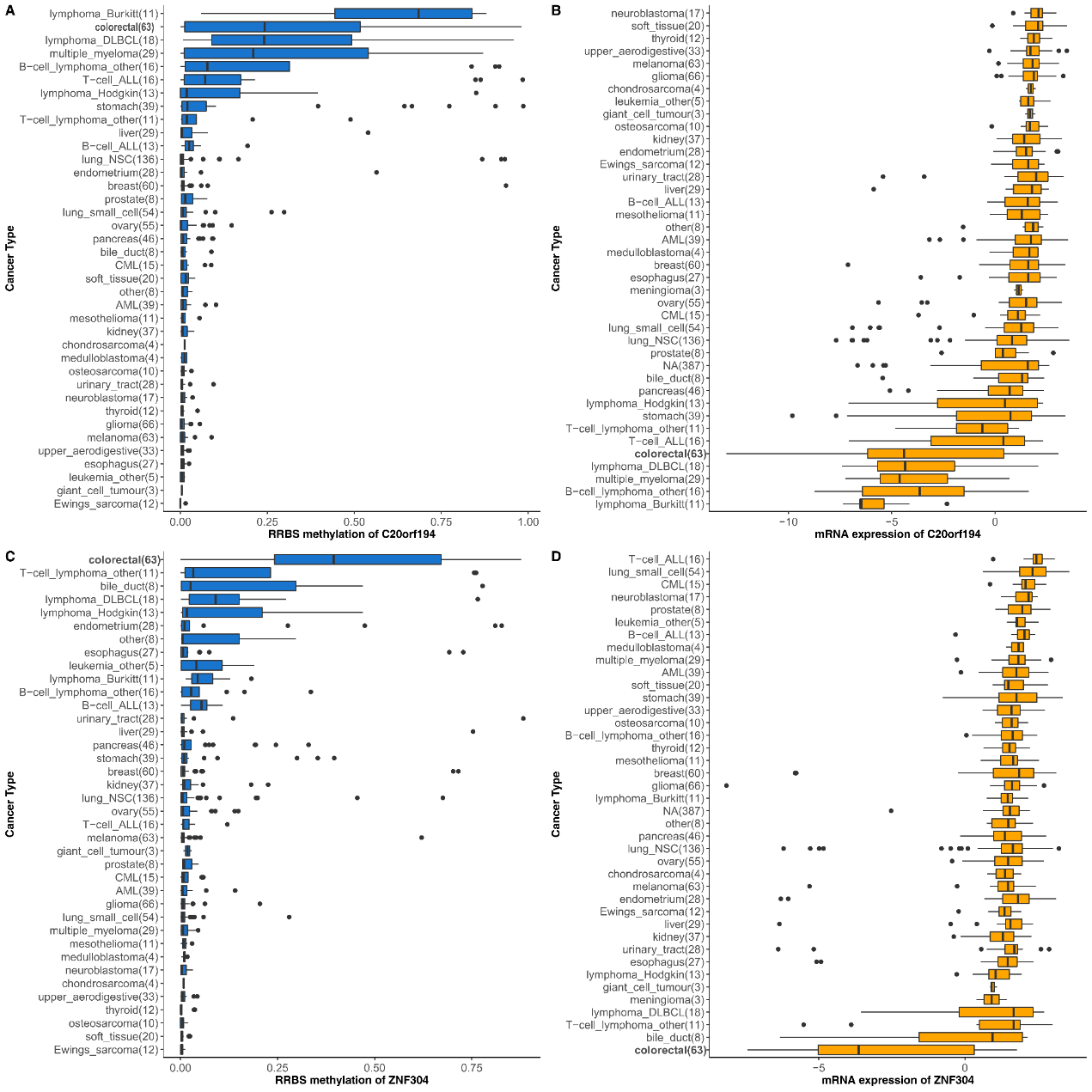


## **Fig. S5. Methylation and expression levels of C20orf194 and ZNF304 in Cancer Cell Line Encyclopedia.**

Relative DNA methylation and mRNA expression levels were extracted from Cancer Cell Line Encyclopedia. Data were sorted by average value of each group. (**A**) Methylation (RRBS) of C20orf194. (**B**) mRNA expression (RNA-seq) of C20orf194. (**C**) Methylation (RRBS) of ZNF304. (**D**) mRNA expression (RNA-seq) of ZNF304.


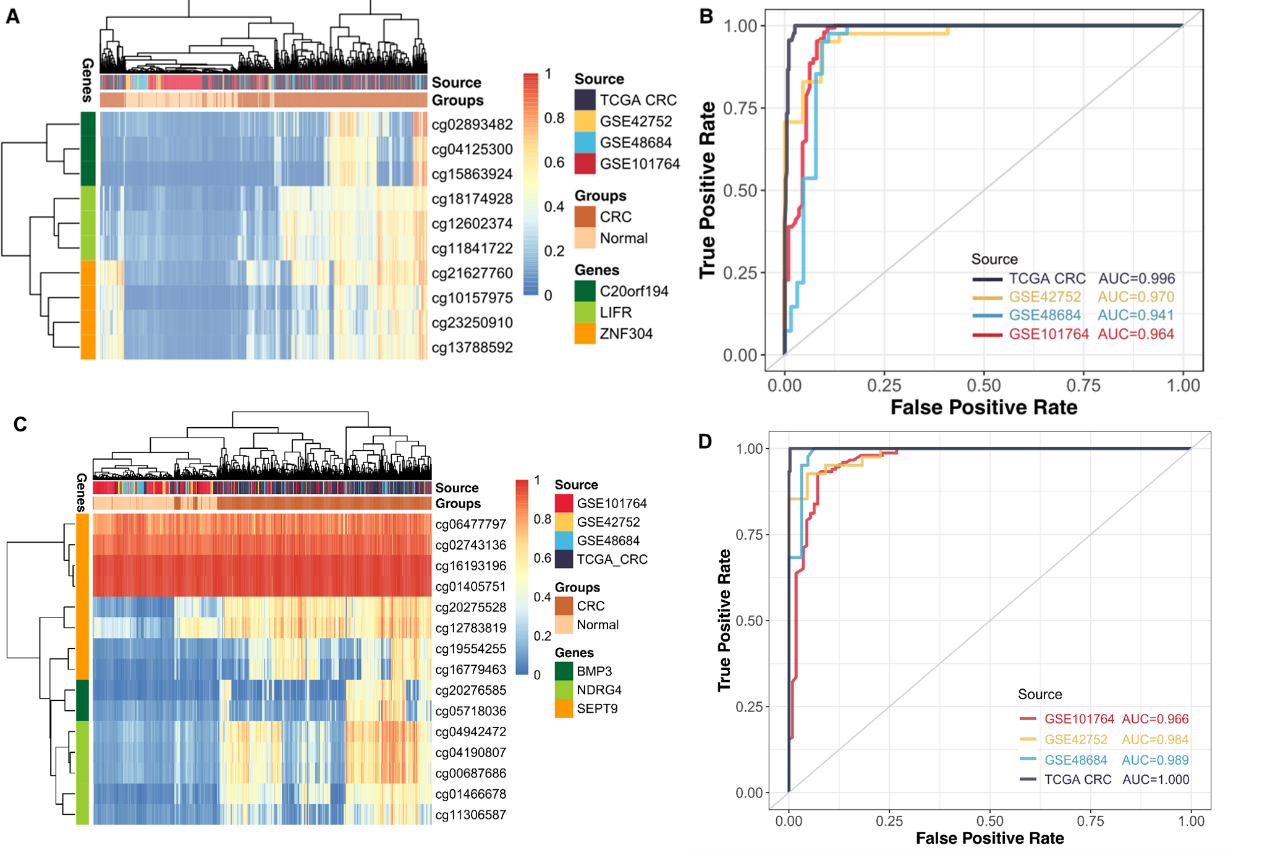


## **Fig. S6. Validation of 10 CRC-specific methylation CpG sites of our study and 15 CpG sites of three commercial biomarkers.**

Unsupervised hierarchical clustering of (**A**) 10 CRC-specific methylation CpG sites of our study and (**C**) 15 CpG sites of three commercial biomarkers for the diagnosis of CRC in four datasets of TCGA CRC, GSE42752, GSE48684, and GSE101764. ROC curves of random forest model using (**B**) 10 CRC-specific methylation CpG sites of our study and (**D**) 15 CpG sites of three commercial biomarkers in four datasets.


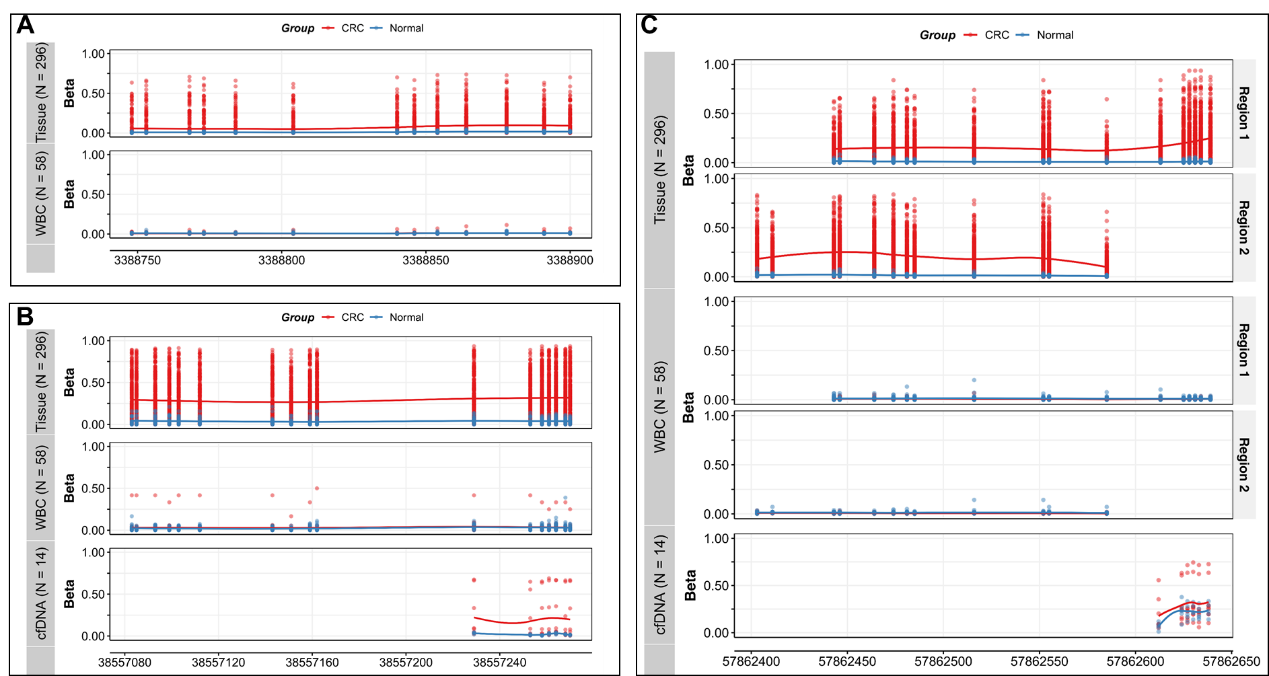


## **Fig. S7. Methylation profiles of (A) C20orf194, (B) LIFR and (C) ZNF304 in an inhouse study using targeted bisulfite sequencing analysis.**

The panel shows the methylation profiles of 272 CRC tissues and 23 normal tissues, white blood cell samples from CRC patients (N = 29) and healthy controls (N = 29), cell-free DNA samples from CRC patients (N = 9) and healthy controls (N = 5). Each dot represents one CpG site for each individual.


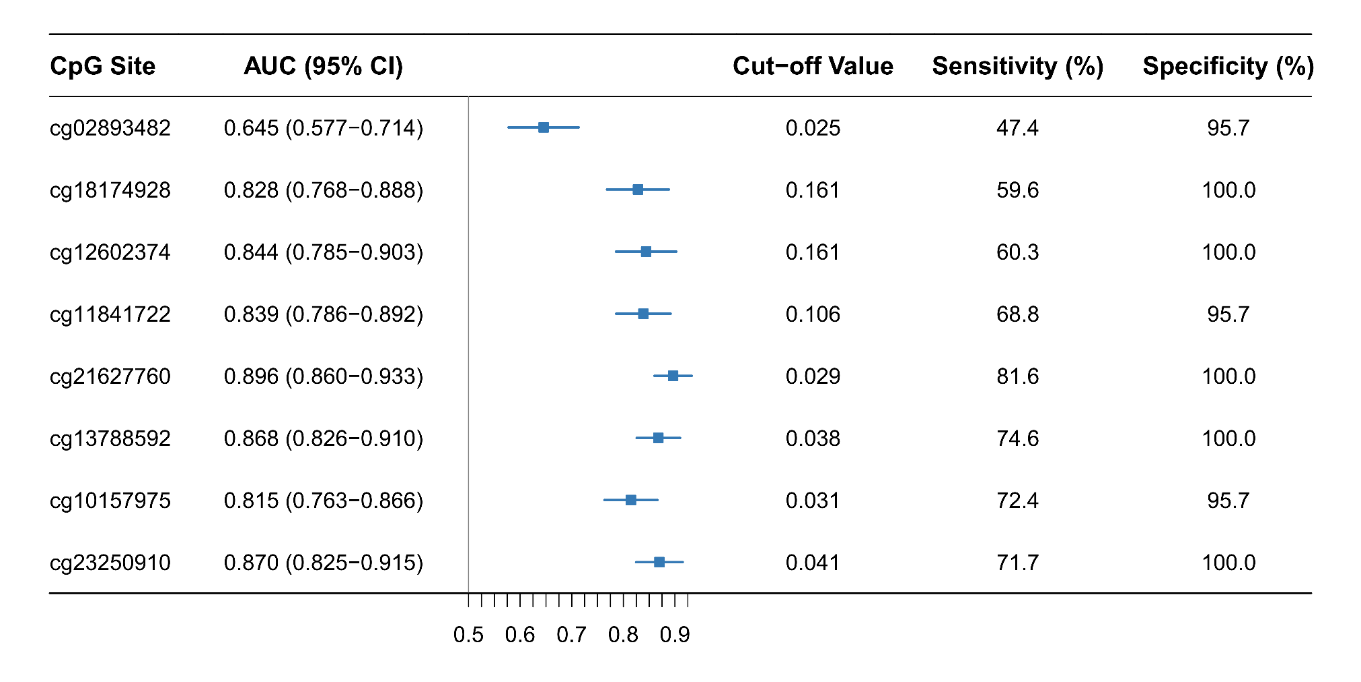


## Fig. S8. Receiver operating characteristic curve analyses of 8 CpG sites for discriminating colorectal cancer tissues and adjacent normal tissues in the inhouse study.
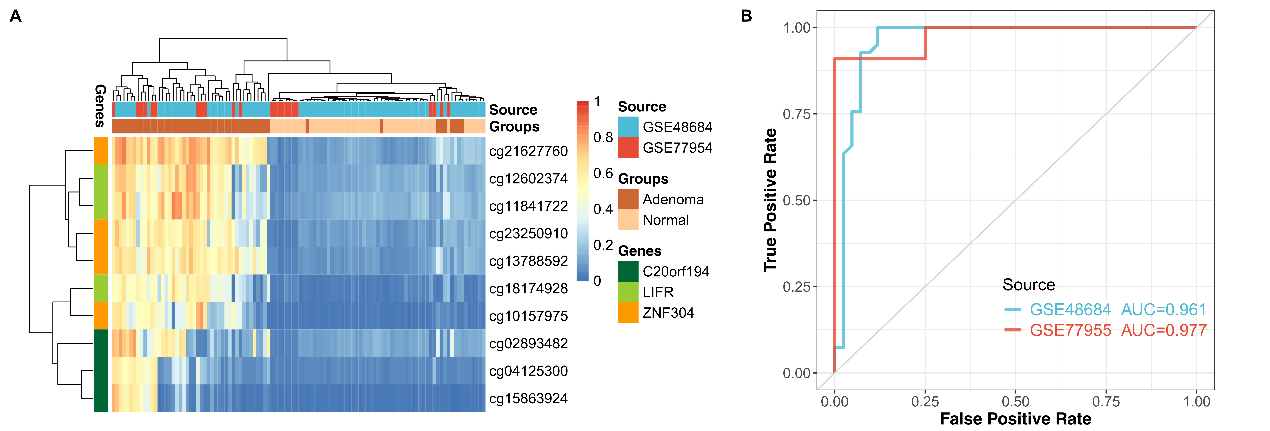


## **Fig. S9. Validation of 10 CRC-specific methylation CpG sites of our study in tissues of colorectal adenomas and normal tissues.**

(**A**) Unsupervised hierarchical clustering of 10 CRC-specific methylation CpG sites of our study in in 2 GEO datasets (GSE48684 and GSE77955) including 54 colorectal adenomas and 52 normal samples. (**B**) ROC curves of random forest model using 10 CRC-specific methylation CpG sites of our study for distinguishing tissues of colorectal adenomas from normal tissues.

# Supplemental Tables

## Table S1. Datasets used for discovery and validation of CRC-specific methylation markers

| **Dataset** | **Source** | **Cancer type** | **Assay** | **Sample type** | **Number of samples** |
| --- | --- | --- | --- | --- | --- |
| **Discovery cohort (N = 5805)** | | | | | |
|  | TCGA | CRC | Infinium 450K | Tissue | Normal = 45, Tumor = 395 |
|  | TCGA | BLCA | Infinium 450K | Tissue | Normal = 21, Tumor = 412 |
|  | TCGA | BRCA | Infinium 450K | Tissue | Normal = 98, Tumor = 785 |
|  | TCGA | ESCA | Infinium 450K | Tissue | Normal = 16, Tumor = 185 |
|  | TCGA | GBM | Infinium 450K | Tissue | Normal = 2, Tumor = 140 |
|  | TCGA | HNSC | Infinium 450K | Tissue | Normal = 50, Tumor = 528 |
|  | TCGA | KIRC | Infinium 450K | Tissue | Normal = 160, Tumor = 319 |
|  | TCGA | LIHC | Infinium 450K | Tissue | Normal = 50, Tumor = 377 |
|  | TCGA | LUAD | Infinium 450K | Tissue | Normal = 32, Tumor = 458 |
|  | TCGA | LUSC | Infinium 450K | Tissue | Normal = 43, Tumor = 372 |
|  | TCGA | UCEC | Infinium 450K | Tissue | Normal = 46, Tumor = 431 |
|  | GSE40279 & GSE69270 | Healthy | Infinium 450K | Blood leukocytes | Normal = 840 |
| **Validation cohort 1 (N = 2045)** | | | | | |
|  | GSE42752 & GSE48684& GSE101764 | CRC | Infinium 450K | Tissue | Normal = 231, Tumor = 198 |
|  | GSE111933 | BLCA | Infinium 450K | Tissue | Normal = 46, Tumor = 46 |
|  | GSE66695 & GSE69914 | BRCA | Infinium 450K | Tissue | Normal = 132, Tumor = 385 |
|  | GSE52826 & GSE79366 | ESCA | Infinium 450K | Tissue | Normal = 10, Tumor = 16 |
|  | GSE36278 & GSE60274 & GSE123678 | GBM | Infinium 450K | Tissue | Normal = 19, Tumor = 210 |
|  | GSE38266 | HNSC | Infinium 450K | Tissue | Normal = 0, Tumor = 42 |
|  | GSE61441 | KIRC | Infinium 450K | Tissue | Normal = 46, Tumor = 46 |
|  | GSE54503 | LIHC | Infinium 450K | Tissue | Normal = 66, Tumor = 66 |
|  | GSE39279 | LUAD | Infinium 450K | Tissue | Normal = 0, Tumor = 322 |
|  | GSE39279 | LUSC | Infinium 450K | Tissue | Normal = 0, Tumor = 122 |
|  | GSE67116 & GSE93589 | UCEC | Infinium 450K | Tissue | Normal = 0, Tumor = 42 |
| **Validation cohort 2 (N = 1457)** | | | | | |
|  | CCLE | 38 cancer types | RRBS | Cell lines | Tumor = 1457 |
| **Validation cohort 3 (N = 353)** | | | | | |
|  | Inhouse study | CRC | Targeted bisulfite sequencing | Tissue | Normal = 23, Tumor = 272 |
|  | Inhouse study | CRC | Targeted bisulfite sequencing | WBC | Normal = 29, Tumor = 29 |
| **cfDNA pilot cohort (N = 14) *** | | | | | |
|  | Inhouse study | CRC | Targeted bisulfite sequencing | cfDNA | Normal = 5, Tumor = 9 |
| **cfDNA validation cohort (N = 155) *** | | | | | |
|  | Inhouse study | CRC | Droplet Digital PCR | cfDNA | Normal =60, Tumor = 95 |

* Nine cfDNA samples from colorectal cancer were overlapped between targeted bisulfite sequencing array and Droplet Digital PCR.

## Table S2. Genomic information of 10 CRC-specific methylation CpG sites of our study and 15 CpG sites from three commercial biomarkers

| **CpG** | **Gene Symbol** | **Gene description** | **Chromosome** | **Genomic Coordinate** | **Strand** | **Relation to Island** | **UCSC RefGene Group** |  |
| --- | --- | --- | --- | --- | --- | --- | --- | --- |
| **Our study** | | | | | | | | |
| cg04125300 | C20orf194 | chromosome 20 open reading frame 194 | 20 | 3388262 | - | Island | TSS200 |  |
| cg15863924 | C20orf194 | chromosome 20 open reading frame 194 | 20 | 3388269 | - | Island | TSS200 |  |
| cg02893482 | C20orf194 | chromosome 20 open reading frame 194 | 20 | 3388900 | + | Island | TSS1500 |  |
| cg18174928 | LIFR | LIF receptor alpha | 5 | 38557085 | + | Island | TSS1500 |  |
| cg12602374 | LIFR | LIF receptor alpha | 5 | 38557162 | + | Island | TSS1500 |  |
| cg11841722 | LIFR | LIF receptor alpha | 5 | 38557253 | - | Island | TSS1500 |  |
| cg23250910 | ZNF304 | zinc finger protein 304 | 19 | 57862410 | - | N_Shore | TSS1500 |  |
| cg10157975 | ZNF304 | zinc finger protein 304 | 19 | 57862442 | + | Island | TSS1500 |  |
| cg13788592 | ZNF304 | zinc finger protein 304 | 19 | 57862612 | - | Island | TSS200 |  |
| cg21627760 | ZNF304 | zinc finger protein 304 | 19 | 57862638 | + | Island | TSS200 |  |
| **Commercial biomarkers *** | | | | | | | | |
| cg20276585 | BMP3 | Bone Morphogenetic Protein 3 | 4 | 81952330 | + | Island | 1stExon |  |
| cg05718036 | BMP3 | Bone Morphogenetic Protein 3 | 4 | 81952593 | + | Island | 1stExon |  |
| cg04190807 | NDRG4 | N-myc downregulated gene 4 | 16 | 58497230 | - | Island | TSS1500 |  |
| cg00687686 | NDRG4 | N-myc downregulated gene 4 | 16 | 58497236 | - | Island | TSS1500 |  |
| cg04942472 | NDRG4 | N-myc downregulated gene 4 | 16 | 58497239 | - | Island | TSS1500 |  |
| cg01466678 | NDRG4 | N-myc downregulated gene 4 | 16 | 58497395 | + | Island | TSS1500 |  |
| cg11306587 | NDRG4 | N-myc downregulated gene 4 | 16 | 58497714 | + | Island | TSS1500 |  |
| cg19554255 | SEPT9 | Septin 9 | 17 | 75369051 | - | Island | TSS1500 |  |
| cg16779463 | SEPT9 | Septin 9 | 17 | 75369055 | - | Island | TSS1500 |  |
| cg20275528 | SEPT9 | Septin 9 | 17 | 75369484 | - | Island | 5'UTR |  |
| cg12783819 | SEPT9 | Septin 9 | 17 | 75369657 | - | Island | 5'UTR |  |
| cg16193196 | SEPT9 | Septin 9 | 17 | 75398290 | + | Island | 5'UTR |  |
| cg01405751 | SEPT9 | Septin 9 | 17 | 75398380 | + | Island | 5'UTR |  |
| cg06477797 | SEPT9 | Septin 9 | 17 | 75398526 | + | Island | 5'UTR |  |
| cg02743136 | SEPT9 | Septin 9 | 17 | 75398583 | + | S_Shore | Body |  |

* CpG sites which were covered by primer sequence of three assays and other published articles were extracted. If primer sequence did not cover a CpG, two surrounding CpG sites were used.

## Table S3. Differential methylation analysis of 10 CRC-specific methylation CpG sites of our study and 15 CpG sites of three commercial biomarkers in CRC samples and normal samples

| **CpG** | **TCGA** | | | | **GSE42752** | | | | **GSE48684** | | | | **GSE101764** | | | | |
| --- | --- | --- | --- | --- | --- | --- | --- | --- | --- | --- | --- | --- | --- | --- | --- | --- | --- |
|  | **CRC** | **Normal** | **Δβ** | ***P*** | **CRC** | **Normal** | **Δβ** | ***P*** | **CRC** | **Normal** | **Δβ** | ***P*** | **CRC** | **Normal** | **Δβ** | ***P*** |  |
| **Our study** | | | | | | | | | | | | | | | | | |
| cg04125300 | 0.229 | 0.033 | 0.195 | 3.92E-47 | 0.24 | 0.071 | 0.169 | 3.33E-03 | 0.229 | 0.051 | 0.177 | 1.37E-07 | 0.22 | 0.069 | 0.151 | 4.45E-11 |  |
| cg15863924 | 0.202 | 0.016 | 0.186 | 4.36E-36 | 0.218 | 0.033 | 0.186 | 4.73E-03 | 0.2 | 0.016 | 0.183 | 6.85E-07 | 0.182 | 0.023 | 0.159 | 1.24E-09 |  |
| cg02893482 | 0.264 | 0.048 | 0.215 | 7.39E-45 | 0.249 | 0.113 | 0.136 | 9.87E-03 | 0.353 | 0.122 | 0.231 | 3.43E-08 | 0.214 | 0.066 | 0.149 | 4.23E-12 |  |
| cg18174928 | 0.353 | 0.024 | 0.328 | 2.60E-113 | 0.337 | 0.084 | 0.253 | 2.49E-06 | 0.289 | 0.032 | 0.257 | 2.11E-14 | 0.317 | 0.067 | 0.251 | 2.10E-23 |  |
| cg12602374 | 0.394 | 0.055 | 0.339 | 7.79E-110 | 0.375 | 0.115 | 0.26 | 7.80E-06 | 0.473 | 0.129 | 0.344 | 6.04E-16 | 0.287 | 0.075 | 0.212 | 7.99E-22 |  |
| cg11841722 | 0.375 | 0.072 | 0.303 | 4.06E-99 | 0.4 | 0.134 | 0.266 | 1.50E-06 | 0.483 | 0.147 | 0.336 | 6.99E-17 | 0.276 | 0.095 | 0.182 | 1.14E-21 |  |
| cg23250910 | 0.338 | 0.048 | 0.29 | 9.88E-89 | 0.331 | 0.073 | 0.258 | 2.40E-06 | 0.36 | 0.103 | 0.257 | 3.08E-11 | 0.235 | 0.053 | 0.183 | 1.36E-23 |  |
| cg10157975 | 0.331 | 0.02 | 0.311 | 4.95E-92 | 0.315 | 0.031 | 0.283 | 5.17E-06 | 0.191 | 0.02 | 0.171 | 4.74E-09 | 0.284 | 0.031 | 0.253 | 5.88E-25 |  |
| cg13788592 | 0.369 | 0.048 | 0.321 | 3.98E-108 | 0.359 | 0.078 | 0.282 | 4.88E-07 | 0.396 | 0.091 | 0.305 | 2.13E-13 | 0.299 | 0.054 | 0.246 | 3.13E-31 |  |
| cg21627760 | 0.475 | 0.082 | 0.393 | 8.34E-98 | 0.433 | 0.095 | 0.337 | 6.15E-08 | 0.462 | 0.118 | 0.343 | 6.14E-14 | 0.368 | 0.074 | 0.294 | 3.25E-33 |  |
| **Commercial biomarkers** | | | | | | | | | | | | | | | | | |
| cg20276585 | 0.239 | 0.015 | 0.224 | 2.21E-52 | 0.21 | 0.036 | 0.174 | 2.42E-03 | 0.231 | 0.018 | 0.213 | 3.17E-09 | 0.206 | 0.034 | 0.172 | 6.24E-13 |  |
| cg05718036 | 0.265 | 0.061 | 0.204 | 1.02E-51 | 0.262 | 0.111 | 0.151 | 2.70E-03 | 0.389 | 0.135 | 0.255 | 5.91E-10 | 0.198 | 0.084 | 0.114 | 7.18E-11 |  |
| cg04190807 | 0.481 | 0.1 | 0.382 | 6.50E-124 | 0.454 | 0.136 | 0.318 | 1.11E-07 | 0.574 | 0.179 | 0.395 | 4.94E-19 | 0.348 | 0.109 | 0.24 | 2.46E-24 |  |
| cg00687686 | 0.474 | 0.096 | 0.378 | 4.48E-110 | 0.483 | 0.145 | 0.338 | 6.88E-08 | 0.558 | 0.193 | 0.365 | 3.61E-18 | 0.351 | 0.107 | 0.244 | 8.00E-24 |  |
| cg04942472 | 0.505 | 0.111 | 0.393 | 2.42E-115 | 0.519 | 0.162 | 0.357 | 1.99E-08 | 0.605 | 0.218 | 0.387 | 1.90E-18 | 0.371 | 0.121 | 0.25 | 6.51E-24 |  |
| cg01466678 | 0.4 | 0.026 | 0.374 | 6.36E-139 | 0.415 | 0.063 | 0.351 | 3.31E-09 | 0.333 | 0.022 | 0.311 | 3.33E-19 | 0.359 | 0.064 | 0.296 | 1.76E-30 |  |
| cg11306587 | 0.291 | 0.043 | 0.247 | 6.05E-75 | 0.343 | 0.091 | 0.252 | 8.36E-06 | 0.237 | 0.047 | 0.191 | 8.04E-14 | 0.305 | 0.087 | 0.218 | 7.63E-22 |  |
| cg19554255 | 0.372 | 0.056 | 0.316 | 1.25E-107 | 0.262 | 0.083 | 0.18 | 2.87E-05 | 0.404 | 0.101 | 0.303 | 4.64E-14 | 0.276 | 0.068 | 0.208 | 1.45E-23 |  |
| cg16779463 | 0.366 | 0.02 | 0.346 | 3.73E-94 | 0.257 | 0.045 | 0.212 | 1.25E-04 | 0.253 | 0.026 | 0.227 | 4.05E-10 | 0.32 | 0.05 | 0.27 | 3.22E-22 |  |
| cg20275528 | 0.598 | 0.121 | 0.478 | 4.86E-33 | 0.507 | 0.104 | 0.404 | 8.90E-14 | 0.494 | 0.1 | 0.394 | 5.06E-25 | 0.533 | 0.144 | 0.39 | 4.53E-59 |  |
| cg12783819 | 0.632 | 0.28 | 0.353 | 2.77E-18 | 0.607 | 0.301 | 0.306 | 1.89E-10 | 0.548 | 0.267 | 0.28 | 4.47E-11 | 0.566 | 0.327 | 0.239 | 1.36E-28 |  |
| cg16193196 | 0.976 | 0.977 | -0.001 | 4.96E-01 | 0.96 | 0.958 | 0.003 | 2.32E-01 | 0.962 | 0.962 | 0 | 9.13E-01 | 0.966 | 0.968 | -0.002 | 1.46E-01 |  |
| cg01405751 | 0.977 | 0.976 | 0.001 | 8.09E-01 | 0.96 | 0.959 | 0.001 | 6.68E-01 | 0.96 | 0.961 | -0.001 | 6.89E-01 | 0.954 | 0.96 | -0.006 | 6.56E-04 |  |
| cg06477797 | 0.869 | 0.883 | -0.015 | 7.99E-04 | 0.863 | 0.864 | -0.001 | 9.31E-01 | 0.927 | 0.927 | 0 | 9.89E-01 | 0.803 | 0.798 | 0.005 | 3.75E-01 |  |
| cg02743136 | 0.929 | 0.915 | 0.014 | 6.59E-05 | 0.91 | 0.907 | 0.005 | 2.94E-01 | 0.914 | 0.919 | -0.005 | 3.11E-01 | 0.88 | 0.882 | -0.003 | 5.08E-01 |  |

## Table S4. Prediction performance of 10 CRC-specific methylation CpG sites of our study and 15 CpG sites of three commercial biomarkers

| **CpG** | **Cut off value** | **AUC** | **TCGA CRC** | | | | **GSE42752** | | | | **GSE48684** | | | | **GSE101764** | | | |
| --- | --- | --- | --- | --- | --- | --- | --- | --- | --- | --- | --- | --- | --- | --- | --- | --- | --- | --- |
|  |  |  | **TP** | **Sen** | **TN** | **Spe** | **TP** | **Sen** | **TN** | **Spe** | **TP** | **Sen** | **TN** | **Spe** | **TP** | **Sen** | **TN** | **Spe** |
| **Our study** | | | | | | | | | | | | | | | | | | |
| cg04125300 | 0.098 | 0.638 | 199 | 0.504 | 45 | 1.000 | 10 | 0.455 | 37 | 0.902 | 29 | 0.453 | 41 | 1.000 | 48 | 0.429 | 140 | 0.940 |
| cg15863924 | 0.072 | 0.651 | 153 | 0.387 | 45 | 1.000 | 10 | 0.455 | 40 | 0.976 | 24 | 0.375 | 41 | 1.000 | 38 | 0.339 | 148 | 0.993 |
| cg02893482 | 0.142 | 0.680 | 183 | 0.463 | 45 | 1.000 | 11 | 0.500 | 36 | 0.878 | 38 | 0.594 | 38 | 0.927 | 46 | 0.411 | 149 | 1.000 |
| cg18174928 | 0.196 | 0.806 | 300 | 0.759 | 45 | 1.000 | 16 | 0.727 | 41 | 1.000 | 39 | 0.609 | 41 | 1.000 | 73 | 0.652 | 149 | 1.000 |
| cg12602374 | 0.172 | 0.843 | 312 | 0.790 | 45 | 1.000 | 16 | 0.727 | 40 | 0.976 | 48 | 0.750 | 40 | 0.976 | 72 | 0.643 | 149 | 1.000 |
| cg11841722 | 0.200 | 0.839 | 307 | 0.777 | 45 | 1.000 | 18 | 0.818 | 39 | 0.951 | 54 | 0.844 | 40 | 0.976 | 70 | 0.625 | 149 | 1.000 |
| cg23250910 | 0.143 | 0.853 | 300 | 0.759 | 44 | 0.978 | 17 | 0.773 | 40 | 0.976 | 42 | 0.656 | 36 | 0.878 | 78 | 0.696 | 149 | 1.000 |
| cg10157975 | 0.086 | 0.836 | 306 | 0.775 | 44 | 0.978 | 17 | 0.773 | 39 | 0.951 | 36 | 0.563 | 41 | 1.000 | 86 | 0.768 | 149 | 1.000 |
| cg13788592 | 0.129 | 0.871 | 328 | 0.830 | 44 | 0.978 | 19 | 0.864 | 39 | 0.951 | 45 | 0.703 | 36 | 0.878 | 92 | 0.821 | 148 | 0.993 |
| cg21627760 | 0.234 | 0.876 | 328 | 0.830 | 45 | 1.000 | 18 | 0.818 | 40 | 0.976 | 43 | 0.672 | 41 | 1.000 | 88 | 0.786 | 149 | 1.000 |
| **Commercial biomarkers** | | | | | | | | | | | | | | | | | | |
| cg20276585 | 0.064 | 0.674 | 203 | 0.514 | 45 | 1.000 | 11 | 0.500 | 38 | 0.927 | 34 | 0.531 | 41 | 1.000 | 51 | 0.455 | 146 | 0.980 |
| cg05718036 | 0.130 | 0.698 | 195 | 0.494 | 45 | 1.000 | 12 | 0.545 | 35 | 0.854 | 57 | 0.891 | 25 | 0.610 | 48 | 0.429 | 148 | 0.993 |
| cg04190807 | 0.197 | 0.884 | 333 | 0.843 | 45 | 1.000 | 20 | 0.909 | 39 | 0.951 | 55 | 0.859 | 34 | 0.829 | 77 | 0.688 | 147 | 0.987 |
| cg00687686 | 0.223 | 0.887 | 310 | 0.785 | 45 | 1.000 | 19 | 0.864 | 39 | 0.951 | 56 | 0.875 | 36 | 0.878 | 73 | 0.652 | 148 | 0.993 |
| cg04942472 | 0.287 | 0.893 | 302 | 0.765 | 45 | 1.000 | 18 | 0.818 | 41 | 1.000 | 49 | 0.766 | 40 | 0.976 | 66 | 0.589 | 149 | 1.000 |
| cg01466678 | 0.197 | 0.914 | 329 | 0.833 | 45 | 1.000 | 20 | 0.909 | 41 | 1.000 | 47 | 0.734 | 41 | 1.000 | 84 | 0.750 | 148 | 0.993 |
| cg11306587 | 0.146 | 0.800 | 271 | 0.686 | 45 | 1.000 | 16 | 0.727 | 40 | 0.976 | 42 | 0.656 | 41 | 1.000 | 75 | 0.670 | 147 | 0.987 |
| cg19554255 | 0.120 | 0.910 | 322 | 0.815 | 44 | 0.978 | 17 | 0.773 | 41 | 1.000 | 52 | 0.813 | 39 | 0.951 | 79 | 0.705 | 147 | 0.987 |
| cg16779463 | 0.084 | 0.818 | 288 | 0.729 | 44 | 0.978 | 14 | 0.636 | 41 | 1.000 | 35 | 0.547 | 40 | 0.976 | 80 | 0.714 | 145 | 0.973 |
| cg20275528 | 0.415 | 0.972 | 373 | 0.944 | 45 | 1.000 | 15 | 0.682 | 41 | 1.000 | 45 | 0.703 | 41 | 1.000 | 97 | 0.866 | 145 | 0.973 |
| cg12783819 | 0.478 | 0.911 | 350 | 0.886 | 40 | 0.889 | 20 | 0.909 | 35 | 0.854 | 43 | 0.672 | 36 | 0.878 | 89 | 0.795 | 125 | 0.839 |
| cg16193196 | 0.974 | 0.669 | 300 | 0.759 | 9 | 0.200 | 1 | 0.045 | 40 | 0.976 | 7 | 0.109 | 38 | 0.927 | 36 | 0.321 | 107 | 0.718 |
| cg01405751 | 0.975 | 0.705 | 314 | 0.795 | 8 | 0.178 | 1 | 0.045 | 40 | 0.976 | 4 | 0.063 | 41 | 1.000 | 1 | 0.009 | 141 | 0.946 |
| cg06477797 | 0.846 | 0.627 | 315 | 0.797 | 3 | 0.067 | 18 | 0.818 | 10 | 0.244 | 64 | 1.000 | 0 | 0.000 | 30 | 0.268 | 141 | 0.946 |
| cg02743136 | 0.930 | 0.711 | 246 | 0.623 | 39 | 0.867 | 3 | 0.136 | 36 | 0.878 | 21 | 0.328 | 34 | 0.829 | 3 | 0.027 | 145 | 0.973 |

TP, True positive; Sen, Sensitivity; TN, True negative; Spe, Specificity.

## Table S5. Misclassification rate of 10 CRC-specific methylation CpG sites of our study and 15 CpG sites of three commercial biomarkers in GEO dataset

| **CpG** | **BLCA** | | **BRCA** | | **ESCA** | | **GBM** | | **HNSC** | | **KIRC** | | **LIHC** | | **LUAD** | | **LUSC** | | **UCEC** | | **Overall (N = 1616)** | | |  |
| --- | --- | --- | --- | --- | --- | --- | --- | --- | --- | --- | --- | --- | --- | --- | --- | --- | --- | --- | --- | --- | --- | --- | --- | --- |
|  | **T** | **N** | **T** | **N** | **T** | **N** | **T** | **N** | **T** | **N** | **T** | **N** | **T** | **N** | **T** | **N** | **T** | **N** | **T** | **N** | **T** | **N** | **T&N** |  |
| **Our study** | | | | | | | | | | | | | | | | | | | | | | | | |
| cg04125300 | 0.000 | 0.000 | 0.000 | 0.000 | 0.000 | 0.000 | 0.000 | 0.000 | 0.143 | NA | 0.000 | 0.000 | 0.106 | 0.000 | 0.050 | NA | 0.025 | NA | 0.000 | NA | 0.061 | 0.049 | 0.059 |  |
| cg15863924 | 0.000 | 0.000 | 0.000 | 0.000 | 0.000 | 0.000 | 0.000 | 0.000 | 0.119 | NA | 0.000 | 0.000 | 0.091 | 0.000 | 0.019 | NA | 0.008 | NA | 0.000 | NA | 0.023 | 0.015 | 0.022 |  |
| cg02893482 | 0.000 | 0.000 | 0.003 | 0.000 | 0.000 | 0.000 | 0.005 | 0.000 | 0.238 | NA | 0.000 | 0.000 | 0.045 | 0.000 | 0.161 | NA | 0.156 | NA | 0.024 | NA | 0.080 | 0.051 | 0.074 |  |
| cg18174928 | 0.109 | 0.000 | 0.003 | 0.000 | 0.000 | 0.000 | 0.000 | 0.000 | 0.000 | NA | 0.000 | 0.000 | 0.061 | 0.000 | 0.003 | NA | 0.025 | NA | 0.000 | NA | 0.042 | 0.024 | 0.038 |  |
| cg12602374 | 0.087 | 0.022 | 0.005 | 0.000 | 0.000 | 0.000 | 0.000 | 0.000 | 0.095 | NA | 0.000 | 0.000 | 0.106 | 0.000 | 0.019 | NA | 0.033 | NA | 0.000 | NA | 0.072 | 0.050 | 0.068 |  |
| cg11841722 | 0.087 | 0.022 | 0.005 | 0.000 | 0.000 | 0.000 | 0.000 | 0.000 | 0.190 | NA | 0.000 | 0.000 | 0.167 | 0.000 | 0.019 | NA | 0.041 | NA | 0.000 | NA | 0.090 | 0.062 | 0.084 |  |
| cg23250910 | 0.000 | 0.000 | 0.000 | 0.000 | 0.000 | 0.000 | 0.000 | 0.000 | 0.262 | NA | 0.022 | 0.000 | 0.015 | 0.000 | 0.031 | NA | 0.033 | NA | 0.071 | NA | 0.054 | 0.033 | 0.050 |  |
| cg10157975 | 0.000 | 0.000 | 0.000 | 0.000 | 0.000 | 0.000 | 0.000 | 0.000 | 0.238 | NA | 0.022 | 0.000 | 0.015 | 0.000 | 0.022 | NA | 0.016 | NA | 0.024 | NA | 0.023 | 0.015 | 0.021 |  |
| cg13788592 | 0.000 | 0.000 | 0.000 | 0.000 | 0.000 | 0.000 | 0.000 | 0.000 | 0.357 | NA | 0.022 | 0.000 | 0.015 | 0.000 | 0.043 | NA | 0.025 | NA | 0.095 | NA | 0.052 | 0.032 | 0.048 |  |
| cg21627760 | 0.000 | 0.000 | 0.000 | 0.000 | 0.000 | 0.000 | 0.000 | 0.000 | 0.214 | NA | 0.022 | 0.000 | 0.015 | 0.000 | 0.034 | NA | 0.016 | NA | 0.095 | NA | 0.064 | 0.041 | 0.059 |  |
| **10 CpG** | **0.109** | **0.022** | **0.062** | **0.068** | **0.000** | **0.000** | **0.000** | **0.000** | **0.452** | **NA** | **0.022** | **0.022** | **0.136** | **0.000** | **0.081** | **NA** | **0.098** | **NA** | **0.095** | **NA** | **0.077** | **0.034** | **0.069** |  |
| **Commercial biomarkers** | | | | | | | | | | | | | | | | | | | | | | | | |
| cg20276585 | 0.000 | 0.022 | 0.364 | 0.030 | 0.688 | 0.000 | 0.448 | 0.000 | 0.524 | NA | 0.065 | 0.000 | 0.348 | 0.015 | 0.146 | NA | 0.115 | NA | 0.286 | NA | 0.097 | 0.025 | 0.083 |  |
| cg05718036 | 0.065 | 0.065 | 0.392 | 0.068 | 0.750 | 0.000 | 0.786 | 0.000 | 0.524 | NA | 0.239 | 0.000 | 0.303 | 0.152 | 0.516 | NA | 0.549 | NA | 0.333 | NA | 0.191 | 0.085 | 0.170 |  |
| cg04190807 | 0.022 | 0.000 | 0.208 | 0.008 | 0.000 | 0.000 | 0.114 | 0.000 | 0.214 | NA | 0.152 | 0.000 | 0.727 | 0.000 | 0.413 | NA | 0.107 | NA | 0.119 | NA | 0.172 | 0.097 | 0.157 |  |
| cg00687686 | 0.022 | 0.000 | 0.182 | 0.000 | 0.000 | 0.000 | 0.095 | 0.000 | 0.238 | NA | 0.130 | 0.000 | 0.500 | 0.000 | 0.332 | NA | 0.074 | NA | 0.119 | NA | 0.171 | 0.101 | 0.157 |  |
| cg04942472 | 0.022 | 0.000 | 0.130 | 0.008 | 0.000 | 0.000 | 0.071 | 0.000 | 0.048 | NA | 0.087 | 0.000 | 0.409 | 0.000 | 0.267 | NA | 0.057 | NA | 0.024 | NA | 0.186 | 0.115 | 0.172 |  |
| cg01466678 | 0.000 | 0.000 | 0.075 | 0.000 | 0.000 | 0.000 | 0.038 | 0.000 | 0.095 | NA | 0.065 | 0.000 | 0.409 | 0.000 | 0.270 | NA | 0.041 | NA | 0.048 | NA | 0.081 | 0.023 | 0.069 |  |
| cg11306587 | 0.022 | 0.000 | 0.060 | 0.000 | 0.000 | 0.000 | 0.014 | 0.000 | 0.167 | NA | 0.087 | 0.000 | 0.545 | 0.000 | 0.199 | NA | 0.016 | NA | 0.000 | NA | 0.096 | 0.054 | 0.087 |  |
| cg19554255 | 0.935 | 0.500 | 0.143 | 0.015 | 0.688 | 0.000 | 0.048 | 0.105 | 0.786 | NA | 0.152 | 0.087 | 0.712 | 0.030 | 0.130 | NA | 0.328 | NA | 0.000 | NA | 0.141 | 0.075 | 0.128 |  |
| cg16779463 | 0.935 | 0.370 | 0.135 | 0.030 | 0.688 | 0.000 | 0.033 | 0.000 | 0.595 | NA | 0.130 | 0.065 | 0.712 | 0.030 | 0.087 | NA | 0.262 | NA | 0.000 | NA | 0.113 | 0.046 | 0.100 |  |
| cg20275528 | 0.891 | 0.087 | 0.184 | 0.008 | 0.688 | 0.000 | 0.000 | 0.000 | 0.310 | NA | 0.109 | 0.000 | 0.591 | 0.015 | 0.065 | NA | 0.230 | NA | 0.000 | NA | 0.156 | 0.063 | 0.137 |  |
| cg12783819 | 0.870 | 0.043 | 0.249 | 0.000 | 0.688 | 0.100 | 0.005 | 0.000 | 0.286 | NA | 0.087 | 0.000 | 0.500 | 0.000 | 0.047 | NA | 0.262 | NA | 0.000 | NA | 0.207 | 0.088 | 0.183 |  |
| cg16193196 | 0.087 | 0.087 | 0.078 | 0.068 | 0.750 | 0.100 | 0.352 | 0.368 | 0.000 | NA | 0.087 | 0.087 | 0.364 | 0.364 | 0.000 | NA | 0.000 | NA | 0.310 | NA | 0.949 | 0.963 | 0.952 |  |
| cg01405751 | 0.022 | 0.022 | 0.008 | 0.000 | 0.625 | 0.200 | 0.114 | 0.368 | 0.000 | NA | 0.435 | 0.457 | 0.303 | 0.333 | 0.000 | NA | 0.000 | NA | 0.214 | NA | 0.943 | 0.960 | 0.946 |  |
| cg06477797 | 0.196 | 0.239 | 0.810 | 0.614 | 0.688 | 0.400 | 0.729 | 0.947 | 0.500 | NA | 0.696 | 0.630 | 0.348 | 0.682 | 0.547 | NA | 0.328 | NA | 0.619 | NA | 0.858 | 0.849 | 0.856 |  |
| cg02743136 | 0.043 | 0.000 | 0.117 | 0.030 | 0.563 | 0.100 | 0.100 | 0.053 | 0.048 | NA | 0.457 | 0.457 | 0.364 | 0.379 | 0.022 | NA | 0.008 | NA | 0.143 | NA | 0.883 | 0.897 | 0.885 |  |
| **15 CpG** | **0.913** | **0.087** | **0.309** | **0.068** | **0.688** | **0.000** | **0.248** | **0.000** | **0.929** | **NA** | **0.217** | **0.000** | **0.833** | **0.167** | **0.705** | **NA** | **0.639** | **NA** | **0.143** | **NA** | **0.493** | **0.075** | **0.410** |  |

T, Tumor; N, Normal

## Table S6. Confusion matrix of random forest model using 15 CpG sites from three commercial biomarkers in distinguishing CRC samples from normal samples

| **Validation dataset** | **TCGA** | | |  | **GSE42752** | | |  | **GSE48684** | | |  | **GSE101764** | | |
| --- | --- | --- | --- | --- | --- | --- | --- | --- | --- | --- | --- | --- | --- | --- | --- |
| **Class** | **True**  **CRC** | **True Normal** | **Totals** |  | **True**  **CRC** | **True Normal** | **Totals** |  | **True**  **CRC** | **True Normal** | **Totals** |  | **True**  **CRC** | **True Normal** | **Totals** |
| Predict CRC | 388 | 0 | 388 |  | 22 | 12 | 34 |  | 64 | 26 | 90 |  | 104 | 13 | 117 |
| Predict Normal | 7 | 45 | 52 |  | 0 | 29 | 29 |  | 0 | 15 | 15 |  | 8 | 136 | 144 |
| Predict Correct | 388 | 45 | 433 |  | 22 | 29 | 51 |  | 64 | 15 | 79 |  | 104 | 136 | 240 |
| Totals | 395 | 45 | 440 |  | 22 | 41 | 63 |  | 64 | 41 | 105 |  | 112 | 149 | 261 |
| Accuracy (95% CI) | 0.984(0.968, 0.994) | | |  | 0.810(0.691, 0.898) | | |  | 0.752(0.659, 0.831) | | |  | 0.920(0.880, 0.950) | | |
| Sensitivity | 0.982 | | |  | 1.000 | | |  | 1.000 | | |  | 0.929 | | |
| Specificity | 1.000 | | |  | 0.707 | | |  | 0.366 | | |  | 0.913 | | |
| Kappa | 0.919 | | |  | 0.628 | | |  | 0.413 | | |  | 0.837 | | |
| Mcnemar's Test P-Value | 2.33E-02 | | |  | 1.50E-03 | | |  | 9.44E-07 | | |  | 3.83E-01 | | |
| Prevalence | 0.898 | | |  | 0.349 | | |  | 0.610 | | |  | 0.429 | | |
| Detection Rate | 0.882 | | |  | 0.349 | | |  | 0.610 | | |  | 0.399 | | |
| Detection Prevalence | 0.882 | | |  | 0.540 | | |  | 0.857 | | |  | 0.448 | | |
| Balanced Accuracy | 0.991 | | |  | 0.854 | | |  | 0.683 | | |  | 0.921 | | |

## Table S7. Sequences of primers and products of targeted bisulfite sequencing array and Droplet Digital PCR

| **Technique** | **Sample type** | **Gene** |  | **Primer** | **Primer Sequence (5'-3')** | **Product information** | | | |
| --- | --- | --- | --- | --- | --- | --- | --- | --- | --- |
|  |  |  |  |  |  | **Genomic Location** | **Product size (bp)** | **Strand** | **CpG** |
| **Targeted bisulfite sequencing array** | | | | | | | | | |
|  | Tissue & WBC | C20orf194 |  | forward | GATTTTAATAGTAAAGGTTAGGGGTTTT | chr20.3388942-3388727 | 216 | - | 13 |
|  |  |  |  | reverse | CCAACRCACCTCCCCAATAC |  |  |  |  |
|  | Tissue & WBC | LIFR |  | forward | GAYGGTTTTGYGGGGAGGA | chr5.38557063-38557297 | 235 | + | 17 |
|  |  |  |  | reverse | CCCCACACCCRACAAAAA |  |  |  |  |
|  | Tissue & WBC | ZNF304 |  | forward | AATTAGAATGTAYGGATYGGGTATAGG | chr19.57862403-57862672 | 270 | - | 16 |
|  |  |  |  | reverse | AAATATCRTTTTCCCAATCTTTTCTC |  |  |  |  |
|  | Tissue & WBC | ZNF304 |  | forward | AYGTTAGAAGTTTYGTTTTTGTAGTTTAGAG | chr19.57862368-57862625 | 258 | - | 12 |
|  |  |  |  | reverse | AAATACATACCCTTTAAAAACAACTATAACC |  |  |  |  |
|  | cfDNA | LIFR |  | forward | AAGAAAATTAGTTTTTGGGAGAG | chr5.38557184-38557291 | 108 | + | 7 |
|  |  |  |  | reverse | ACCCRACAAAAAAACCAAAA |  |  |  |  |
|  | cfDNA | ZNF304 |  | forward | AATGATAGTTTAGAAATTGGGYG | chr19.57862563-57862671 | 109 | + | 6 |
|  |  |  |  | reverse | CCAAAATACACRAACCRAACATAA |  |  |  |  |
| **Droplet Digital PCR** | | | | | | | | | |
|  | cfDNA | LIFR | Reaction mixture1 | Methylated forward | GGGATTTTGCGAATTATTTAAATAG | chr5.38557220-38557285 | 66 | + | 7 |
|  |  |  |  | Methylated reverse | CAAAAAAACCAAAACGCGAA |  |  |  |  |
|  |  |  |  | Methylated probe | FAM-ACGACGCCTCGAC-MGB |  |  |  |  |
|  |  |  |  | Unmethylated forward | TGATTGTTGGGATTTTGTGA | chr5.38557212-38557291 | 80 | + | 0 |
|  |  |  |  | Unmethylated reverse | ACCCAACAAAAAAACCAAAAC |  |  |  |  |
|  |  |  |  | Unmethylated probe | VIC-CAACACCTCAACCC-MGB |  |  |  |  |
|  |  |  | Reaction mixture2 | Universal forward | TGTTGGGATTTTGYGAATTATTTA | chr5.38557216-38557286 | 71 | + | - |
|  |  |  |  | Universal reverse | ACAAAAAAACCAAAACRCRAAC |  |  |  |  |
|  |  |  |  | Methylated probe | FAM-ACGACGCCTCGAC-MGB |  |  |  |  |
|  |  |  |  | Unmethylated probe | VIC-CAACACCTCAACCC-MGB |  |  |  |  |
| ` | cfDNA | ZNF304 | Reaction mixture1 | Methylated forward | GTTGTAGGGGCGAGATTT | chr19.57862602-57862670 | 69 | + | 8 |
|  |  |  |  | Methylated reverse | CAAAATACACGAACCGAACATA |  |  |  |  |
|  |  |  |  | Methylated probe | FAM-AATACGTCACGACGACGACGCCA-BHQ1 |  |  |  |  |
|  |  |  |  | Unmethylated forward | GTTGTTTTGGGTTGTAGGG | chr19.57862592-57862675 | 84 | + | 0 |
|  |  |  |  | Unmethylated reverse | ACAACCAAAATACACAAACCA |  |  |  |  |
|  |  |  |  | Unmethylated probe | VIC-CATCACAACAACAACA-MGB |  |  |  |  |
|  |  |  | Reaction mixture2 | Methylated forward | GTTGTAGGGGCGAGATTT | chr19.57862602-57862671 | 70 | + | 8 |
|  |  |  |  | Universal reverse | CCAAAATACACRAACCRAACATA |  |  |  |  |
|  |  |  |  | Methylated probe | FAM-AATACGTCACGACGACGACGCCA-BHQ1 |  |  |  |  |
|  |  |  |  | Unmethylated forward | GTTGTTTTGGGTTGTAGGG | chr19.57862592-57862671 | 80 | + | 0 |
|  |  |  |  | Universal reverse | CCAAAATACACRAACCRAACATA |  |  |  |  |
|  |  |  |  | Unmethylated probe | VIC-CATCACAACAACAACA-MGB |  |  |  |  |
